# Endothelial KLF11 is a novel protector against diabetic atherosclerosis

**DOI:** 10.1186/s12933-024-02473-y

**Published:** 2024-10-26

**Authors:** Guizhen Zhao, Yang Zhao, Wenying Liang, Haocheng Lu, Hongyu Liu, Yongjie Deng, Tianqing Zhu, Yanhong Guo, Lin Chang, Minerva T. Garcia-Barrio, Y. Eugene Chen, Jifeng Zhang

**Affiliations:** 1 Frankel Cardiovascular Center, Department of Internal Medicine, University of Michigan Medical Center, Ann Arbor, MI 48109 USA; 2 Department of Pharmacological and Pharmaceutical Sciences, University of Houston College of Pharmacy, Houston, TX 77204 USA; 3 Division of Rheumatology, Department of Internal Medicine, University of Michigan Medical Center, Ann Arbor, MI 48109 USA; 4 School of Medicine, Southern University of Science and Technology, Shenzhen, 518055 People’s Republic of China

**Keywords:** Atherosclerosis, Diabetes, Endothelial cell, KLF11, Endothelial-to-mesenchymal transition (EndMT)

## Abstract

**Background:**

Atherosclerotic cardiovascular diseases remain the leading cause of mortality in diabetic patients, with endothelial cell (EC) dysfunction serving as the initiating step of atherosclerosis, which is exacerbated in diabetes. Krüppel-like factor 11 (KLF11), known for its missense mutations leading to the development of diabetes in humans, has also been identified as a novel protector of vascular homeostasis. However, its role in diabetic atherosclerosis remains unexplored.

**Methods:**

Diabetic atherosclerosis was induced in both EC-specific KLF11 transgenic and knockout mice in the Ldlr^−/−^ background by feeding a diabetogenic diet with cholesterol (DDC). Single-cell RNA sequencing (scRNA-seq) was utilized to profile EC dysfunction in diabetic atherosclerosis. Additionally, gain- and loss-of-function experiments were conducted to investigate the role of KLF11 in hyperglycemia-induced endothelial cell dysfunction.

**Results:**

We found that endothelial KLF11 deficiency significantly accelerates atherogenesis under diabetic conditions, whereas KLF11 overexpression remarkably inhibits it. scRNA-seq profiling demonstrates that loss of KLF11 increases endothelial-to-mesenchymal transition (EndMT) during atherogenesis under diabetic conditions. Utilizing gain- and loss-of-function approaches, our in vitro study reveals that KLF11 significantly inhibits EC inflammatory activation and TXNIP-induced EC oxidative stress, as well as Notch1/Snail-mediated EndMT under high glucose exposure.

**Conclusion:**

Our study demonstrates that endothelial KLF11 is an endogenous protective factor against diabetic atherosclerosis. These findings indicate that manipulating KLF11 could be a promising approach for developing novel therapies for diabetes-related cardiovascular complications.

**Supplementary Information:**

The online version contains supplementary material available at 10.1186/s12933-024-02473-y.

## Background

Accelerated atherosclerosis in patients with diabetes is a major cause of morbidity and mortality [1, 2]. Diabetes is also recognized as an independent risk factor for atherosclerosis, and their pathological link has been demonstrated in clinical settings [2, 3]. Human genetic studies have revealed an association between the gene polymorphisms of Krüppel-like factor 11 (*KLF11*), a member of the Sp1/KLF family of zinc finger transcription factors, and diabetes [4]. Specifically, missense mutations in the human *KLF11* lead to maturity-onset diabetes of the young type 7 (MODY7), an early-onset type 2 diabetes, and its monogenic mutation causes early childhood-onset type 1B diabetes [4, 5]. Additionally, high glucose concentration induces KLF11 expression in β-cells, and KLF11 protein can directly activate insulin gene expression [4, 6, 7]. Notably, *Klf11* null mice do not develop diabetes but still show decreased circulating insulin, abnormal glucose tolerance, and increased insulin sensitivity in peripheral organs [8]. Moreover, KLF11 has been identified as an essential regulator of hepatic triglyceride metabolism by increasing fatty acid oxidation [9]. These findings underscore the crucial role of KLF11 in the regulation of metabolic gene networks. However, its specific role in atherosclerosis under diabetic conditions remains unexplored.

Endothelial cell (EC) dysfunction is a pivotal feature shared by both diabetes and atherosclerosis, playing a central role in the onset and progression of atherosclerosis in murine models of diabetes and dyslipidemia [10–12]. In humans, the coexistence of dyslipidemia and hyperglycemia accelerates atherogenesis, highlighting EC dysfunction as an initial trigger of atherosclerosis in diabetic patients. Hyperglycemia, a hallmark of diabetes, has been identified as a primary driver of EC dysfunction in diabetic atherogenesis [11, 13]. EC dysfunction is characterized by reduced nitric oxide bioavailability, increased inflammation, oxidative stress, and abnormal secretion of various vasoactive factors. These changes lead to increased vascular permeability, recruitment of leukocytes, and remodeling of the extracellular matrix, all of which collectively contribute to the development of atherosclerosis [14, 15]. We and others have demonstrated the protective role of endothelial KLF11 in maintaining vascular homeostasis in various diseases, including sepsis, stroke, abdominal aortic aneurysm (AAA), thrombosis, and renal injury [16–21]. In particular, our previous study identified the anti-inflammatory and antioxidant effects of KLF11 in aortic ECs in response to tumor necrosis factor-α (TNF-α) and angiotensin II [20]. Despite these insights, the potential function of KLF11 in diabetic endothelium and the underlying mechanisms remain undiscovered.

In this study, we found that endothelial KLF11 deficiency significantly aggravates atherosclerotic lesion formation in diabetic *Ldlr*^−/−^ mice. Using in vitro gain- and loss-of-function approaches, we systemically demonstrated that KLF11 plays a pivotal role in safeguarding EC dysfunction in response to high glucose by inhibiting TXNIP-induced EC oxidative stress and preventing Notch1/Snail-mediated endothelial to mesenchymal transition (EndMT). Our discoveries reveal promising new pathways for innovative therapeutic strategies for diabetic atherosclerosis.

## Methods

### Materials and reagents

KLF11 antibody (X1710) was purchased from the Syd Labs (Natick, MA). CD31 antibody (DIA-310) was from Dianova (Hamburg, Germany). The antibodies for Galectin 3 (Mac2) (14-5301-85) and TXNIP (40-3700) were from ThermoFisher Scientific (Waltham, MA). The antibodies for VCAM1 (sc-13160), E-selectin (sc-14011) and EIF5 (sc-28309) were from Santa Cruz Biotechnology (Santa Cruz, CA). Antibodies for smooth muscle α-actin (ab119952) and SM22α (ab103135) were from Abcam (Boston, MA). Antibodies for β-actin (4967), Slug (9585), Flag tag (14793), Di-Methyl-Histone H3 (Lys9) (H3K9me2, 4658), Tri-Methyl-Histone H3 (Lys27) (H3K27me3, 9733) and rabbit IgG (2729) were from Cell Signaling Technology (CST, Danvers, MA). DLL4 antibody (A12943) was from Abclonal (Woburn, MA). D-glucose (G7021), L-glucose (G5500), and Lipopolysaccharide (LPS) from *Escherichia coli* (L4391) were purchased from Millipore-Sigma (St. Louis, MO). The 5,5-Dimethyl-1-Pyrroline-N-Oxide (DMPO, 3317-61-1) was from Cayman Chemical (Ann Arbor, MI). MitoSOX™ Red mitochondrial superoxide indicators (M36007) and MitoTracker™ Green FM (M7514) were from ThermoFisher Scientific (Waltham, MA). The 8-oxo-dG antibody (4354-MC-050) was from R&D Systems.

### Human aortic samples

Detailed information on human aortic tissues from patients or organ donors is listed in Online Table I. For additional information, see ‘Ethics declaration’.

### Animals

EC-specific *Klf11*-knockout (Tie2Cre^+^-*Klf11*^f/f^) and transgenic (Tie2-Klf11-Tg) mice, as we previously reported [20], were crossbred with *Ldlr*^−/−^ mice (The Jackson Laboratory, stock No. 002207) to generate *Klf11*^ECKO^-*Ldlr*^−/−^ and *Klf11*^ECtg^-*Ldlr*^−/−^ mice used for this study.

### Diabetic atherosclerosis model and analysis of atherosclerosis in mice

Diet-induced diabetic atherosclerosis was performed as previously described [22]. Briefly, 8-week-old *Klf11*^ECKO^-*Ldlr*^−/−^, *Klf11*^ECtg^-*Ldlr*^−/−^ and their littermate controls (both males and females) were given a diabetogenic diet with 0.15% cholesterol (DDC; Envigo, TD.180368, a diet formulated with 37.7% carbohydrate, 35.7% fat as lard, and 17.7% protein, by weight, with 0.15% cholesterol added) for 12 weeks or 16 weeks. The DDC provides 35.7% calories from fat and 37.8% from carbohydrates. The total number of mice required for each group was estimated based on our previous experience, power calculation, and expected differences in measurements to be performed. Using the VINPEPI statistical program [23], assuming α error rate of 0.05, β error rate of 0.20, and 25% difference, and adding two extra mice per group to control for accidental loss (attrition rate 20%), the number of mice per sex and per group is *n* = 10.

At the endpoint, animals were fasted for 4 h and then anesthetized by an overdose of CO_2_. As previously described, two methods were used to analyze the atherosclerotic lesions [24, 25]. All analyses were conducted in a blinded fashion and following the American Heart Association recommendations for experimental atherosclerosis studies [26]. For *en face* analysis of the lesions in the whole aortic tree, the entire aorta from the heart to the iliac bifurcation was isolated. After clearing the perivascular adipose and adventitial tissue, aortas were stained with Oil Red O (ORO) solution [1.6 mg/ml ORO powder from Sigma, 77.78% (v/v) methanol, and 40 mg/ml NaOH] for 50 min, followed by incubation in 70% ethanol for 30 min and washing once with distilled water. The stained aortas were opened longitudinally, and the percentage of plaque area stained by ORO to the total luminal surface area was quantified using ImageJ (National Institutes of Health, NIH).

### Pathological analysis of atherosclerotic lesions in the aortic sinus

Six locations (separated by 50 μm) of the cross-sections from the aortic sinus were examined, and the largest plaques of the 3 valves were used for morphological analysis. An average of 3 sections of each animal were used for quantification.

Formalin-fixed aortic roots were cryoprotected in 40% sucrose overnight at 4 °C, embedded in O.C.T compound (Tissue-Tek, 4583), and snap-frozen in liquid nitrogen. The frozen blocks were sectioned at 7 microns on a Leica Cryostat (Leica, CM3050S), and the slides were used for the examinations below.

ORO staining of slides was performed using commercial ORO staining solution (Sigma-Aldrich, 1.02419) according to the manufacturer’s recommendation. Briefly, slides were immersed in 60% 2-propanol for 20 s and transferred to ORO staining solution for 10 min, 60% 2-propanol for 30 s, and then washed with distilled water for 20 s. Finally, the slides were stained with hematoxylin (ThermoFisher Scientific, 842) solution and mounted with Aquatex (Sigma-Aldrich, 108562).

Masson Trichrome staining was performed by the ULAM Pathology Core at the University of Michigan. For immunofluorescence staining, the slides were blocked in 5% donkey serum for 1 h at room temperature and then incubated with Mac2 antibody (ThermoFisher Scientific, 14-5301-85) or species-matched IgG (Santa Cruz, sc-2026) as negative control at 4 °C overnight. After washing with PBS for 3 times, the slides were incubated with Alexa Fluor-conjugated secondary antibodies (Jackson ImmunoResearch Laboratories) for 1 h at room temperature, washed with PBS, and then mounted with ProLong™ Gold Antifade Mountant with DAPI (Invitrogen, P36935).

Images were captured with an Olympus DP73 microscope. The total plaque area, necrotic core (selection anuclear, fibrotic area) area, and percentages of collagen deposition and Mac2 + area in the plaques were quantified with ImageJ software.

### Oral glucose tolerance (OGTT) and insulin tolerance test (ITT)

For OGTT, mice were fasted overnight. Next, glucose was administered (2 g/kg, gavage), and blood glucose levels were measured at the indicated time points thereafter. For ITT, mice were injected intraperitoneally with human insulin (Humulin R, 1 U/kg) following 6 h of fasting. Blood glucose levels were measured at the indicated time points after insulin administration.

### En face immunofluorescence staining

Male *Ldlr*^−/−^ mice at 10 weeks old were fed a Chow diet (CD) or DDC for 12 weeks. After perfusion and fixation, the ascending thoracic aortas from these mice were isolated and incubated in PBS with 5% normal donkey serum for 30 min while rocking at room temperature. The rabbit anti-KLF11 antibody (Syd labs, X1710, 1:50 dilution) and rat anti-CD31 (Dianova, DIA-310, 1:100 dilution), or rabbit or rat IgG were incubated with the aortas overnight with rocking at 4 °C. Alexa Fluor-conjugated secondary antibodies (Jackson ImmunoResearch Laboratories) were applied for 1 h with rocking at room temperature to label primary antibodies. After washing 3 times with PBS, the aortas were longitudinally opened to expose the endothelium. The aortas were mounted on the cover glasses using ProLong^™^ Gold Antifade Mountant with DAPI (Invitrogen, P36935), with the endothelium facing down. Immunofluorescence images were captured with a Nikon A1 inverted confocal microscope.

### Pathological analysis of atherosclerotic lesions in the aortic sinus

Formalin-fixed aortic roots were cryoprotected in 40% sucrose overnight at 4 °C, embedded in O.C.T compound (Tissue-Tek, 4583), and snap-frozen in liquid nitrogen. The frozen blocks were sectioned at 7 microns on a Leica Cryostat (Leica, CM3050S), and the slides were used for the examinations below.

ORO staining of slides was performed using commercial ORO staining solution (Sigma-Aldrich, 1.02419) according to the manufacturer’s recommendation. Briefly, slides were immersed in 60% 2-propanol for 20 s and transferred to ORO staining solution for 10 min, 60% 2-propanol for 30 s, and then washed with distilled water for 20 s. Finally, the slides were stained with hematoxylin (ThermoFisher Scientific, 842) solution and mounted with Aquatex (Sigma-Aldrich, 108562).

Masson Trichrome staining was performed by the In-Vivo Animal Core at the University of Michigan.

For immunofluorescence staining, the slides were blocked in 5% donkey serum for 1 h at room temperature and then incubated with Mac2 antibody (ThermoFisher Scientific, 14-5301-85) or species-matched IgG (Santa Cruz, sc-2026) as negative control at 4 °C overnight. After washing with PBS for 3 times, the slides were incubated with Alexa Fluor-conjugated secondary antibodies (Jackson ImmunoResearch Laboratories) for 1 h at room temperature, washed with PBS, and then mounted with ProLong™ Gold Antifade Mountant with DAPI (Invitrogen, P36935).

Images were captured with an Olympus DP73 microscope. The total plaque area, necrotic core (selection anuclear, fibrotic area) area, and percentages of collagen deposition and Mac2 + area in the plaques were quantified with ImageJ software.

### In vivo leukocyte adhesion by intravital microscopy analysis

Intravital microscopy analysis was performed as described before [19, 27]. Briefly, 10-week-old male *Klf11*^ECKO^ and *Klf11*^f/f^ mice were intraperitoneally administered lipopolysaccharide (LPS, 50 µg/kg). After 4 h, rhodamine 6G (0.3 mg/kg, Sigma Chemical, 83697) was infused by tail vein injection to label leukocytes 30 min before the intravital microscopy procedure. After anesthetization, the cremaster muscle of mice was dissected from the surrounding tissues. The muscle was cut longitudinally and kept flat against the pedestal by fixing the edges via silk sutures. The muscle was then superfused with saline at 37 °C, and intravital microscopy was used to visualize the cremasteric microcirculation and track the leukocyte movement. Leukocytes remaining stationary for more than 30 s were considered as adhesion to endothelium. Adherent leukocytes were counted as the number per 100 mm length of venule.

### Cell culture

Human coronary artery endothelial cells (HCAECs, CC-2585) from a 40-year-old male were purchased from Lonza (Walkersville, MD) and cultured in endothelial cell growth media-2 (EGM-2, CC-3202, Lonza, Walkersville, MD) at 37 °C, 5% CO_2_ in a humidified cell culture incubator. HCAECs from passages 4–8 were used in all experiments. The human monocyte cell line THP-1 cells were purchased from ATCC (Manassas, VA) and grown in RPMI 1640 containing 10% FBS (ThermoFisher Scientific, Waltham, MA) and 50 mg/ml of a penicillin/streptomycin mix.

### siRNA transfection

HCAECs were transfected with 30nM si*KLF11* (ThermoFisher Scientific, s13158), si*TXNIP* (ThermoFisher Scientific, 135850), or Silencer™ negative control siRNA (siControl, ThermoFisher Scientific, 4390843) using Lipofectamine RNAiMAX (Invitrogen, 13778150) according to the manufactures’ recommendation.

### Total RNA isolation and quantitative real-time PCR analysis

Total RNA from tissue was purified using TRIzol (Invitrogen, 15596018). Total RNA from cultured cells was extracted using RNeasy Mini Kit (QIAGEN, 74106, Hilden, Germany) according to the manufacturer’s instructions. SuperScript™ III First-Strand Synthesis System (ThermoFisher Scientific, 18080051) and random primers were used to reverse transcribe RNA into cDNA. Gene expression was quantified by Real-Time PCR Detection System (BioRad, Hercules, CA) using SYBR Green Fast qPCR Mix (Abclonal, RK21203). The gene expression level was normalized to the internal control PPIA (peptidylprolyl isomerase A). The used qPCR primers are listed in Online Table II.

### Protein extraction and western blot

Tissues or cells were lysed in RIPA lysis buffer (ThermoFisher Scientific, 89901, Waltham, MA) supplemented with the cOmplete™ EDTA-free protease inhibitor cocktail (Roche, 11873580001, Penzberg, Germany) and PhosSTOP™ phosphatase inhibitor (Roche, 4906845001, Penzberg, Germany). Protein extracts were resolved in SDS-PAGE gels and transferred to nitrocellulose membranes (BioRad, 1620115, Hercules, CA). Membranes were blocked in 5% fat-free milk for 1 h at room temperature and incubated with primary antibodies at 4 °C overnight. After washing three times with 1xTBST, membranes were incubated with a secondary antibody (1:10000 dilution, Li-Cor Bioscience) for 1 h at room temperature. After three washes with 1xTBST, bands were scanned using the Odyssey Imaging System (Li-Cor Bioscience) and quantified with the LI-COR Image Studio Software.

### In vitro leukocyte-endothelial adhesion assay

HCAECs were infected with Ad-*lacZ*, Ad-*KLF11* or Ad-sh-*KLF11*. After 24 h, HCAECs were cultured in growth media with normal glucose (5 mM D-glucose, NG) or high glucose (25 mM D-glucose, HG) for 24 h and subsequently incubated for 30 min with THP-1 cells, which were pre-stained with 1 μm Calcein A/M. The unbound THP-1 cells were removed by three washes with PBS. The adhered cells were fixed with 4% paraformaldehyde, visualized using fluorescence microscopy, and adherence was calculated from 9 random fields per well using Image J software.

### Cellular ROS/superoxide detection assay

Total ROS and superoxide produced in HCAECs were measured using the Cellular ROS/Superoxide Detection Assay Kit (Abcam, ab139476), following the manufacturer’s protocol.

### In vitro immuno-spin trapping of free radicals

Immuno-spin trapping of cellular free radicals was performed as previously described [28, 29]. Briefly, HCAECs were transfected with siControl, siKLF11, or si*TXNIP*. After 48 h, the cells were treated with 25mM D-glucose, 2ng/ml TNF-α, and 25mM DMPO (Cayman Chemical, 10006436) for 2 h. Next, the cells were fixed in 4% paraformaldehyde for 15 min, permeabilized in permeabilizing solution (0.1% Triton X-100 and 0.05% BSA) for 10 min and blocked in 5% donkey serum for 1 h at room temperature. The rabbit anti-DMPO (1:50, US Biological, D8075-94 A) and goat anti-VE-cadherin (1:50, Santa Cruz Biotechnology, sc-6458) antibodies were incubated with cells overnight at 4 °C. After washing with PBS, the cells were incubated with Alexa Fluor-conjugated secondary antibodies (Jackson ImmunoResearch Laboratories), washed, and subsequently mounted with ProLong^™^ Gold Antifade Mountant with DAPI (Invitrogen, P36935) before images were captured with an Olympus IX73 microscope.

### Mitochondrial ROS staining

HCAECs were transfected with siControl, or siKLF11. After 48 h, the cells were treated with 25mM D-glucose for 2 h, followed by staining with MitoSOX™ Red (Invitrogen, M36008) and MitoTracker™ Green FM (Invitrogen, M7514) for 30 min, following the manufacturer’s instructions. Cells were fixed with 4% paraformaldehyde and subsequently mounted with ProLong™ Gold Antifade Mountant with DAPI (Invitrogen, P36935) before images were captured with a KEYENCE BZ-X800 All-in-one Fluorescence Microscope. Excitation/emission wavelengths of 396/610nm were used following manufacturer’s instruction to detect mitochondrial superoxide specifically.

### Oxidative DNA damage detection

HCAECs were transfected with siControl, or siKLF11. After 48 h, the cells were treated with 25mM D-glucose for 2 h. Cell were fixed with 1:1 methanol, acetone for 20 min at -20 °C, treated with 0.05 N HCl for 5 min at 4 °C and incubated with 100ug/ml RNAse in 150mM NaCl, 25mM sodium citrate for 1 h at 37 °C. After washing sequentially in PBS, 35%, 50% and 75% ethanol, for 3 min each, cells were incubated in 0.15 N NaOH in 70% ethanol for 4 min to denature DNA. After washing sequentially in 70% ethanol containing 4% formaldehyde, 50% and 35% ethanol, and PBS for 2 min each, cells were incubated in 5ug/ml proteinase K in 20 Mm Tris (pH7.5) and 1 Mm EDTA for 10 min at 37 °C. After blocking in 5% donkey serum for 1 h at room temperature, the cells were incubated with mouse 8-oxo-dG antibody (1:100, R&D Systems, 4354-MC-050) and rabbit anti-VE-cadherin (1:200, Cell Signaling Technology, 2500 S) antibodies overnight at 4 °C. After washing with PBS, the cells were incubated with Alexa Fluor-conjugated secondary antibodies (Jackson ImmunoResearch Laboratories) and subsequently mounted with ProLong™ Gold Antifade Mountant with DAPI (Invitrogen, P36935) before images were captured with a KEYENCE BZ-X800 All-in-one Fluorescence Microscope.

### Chromatin immunoprecipitation (ChIP) assay

ChIP assays were performed using the SimpleChIP Enzymatic Chromatin IP kit (Magnetic Beads) (CST, 9003S), according to the manufacturer’s protocol. In brief, HCAECs were infected with Ad-*lacZ* or Ad-flag-*KLF11*. After 48 h, the cells were treated with 1% formaldehyde for 10 min at room temperature for cross-linking, and these reactions were stopped by adding 0.1% glycine. Cells were lysed, and chromatin extracts were digested with Micrococcal Nuclease at 37°C for 20 min, followed by sonication (Branson Sonifier SLPe, 10 seconds of 35% amplification, three times). After centrifugation, the chromatin supernatants were incubated with anti-flag antibody (1:100, CST, 14793S) or normal rabbit IgG (CST, 2729) at 4°C overnight with gentle rotation. The DNA/protein complexes were immunoprecipitated by ChIP grade protein G magnetic beads with rotation for 2 h at 4°C, followed by three washes in low-salt buffer, one wash in high salt buffer, and elution at 65°C for 30min. The cross-linking of the eluted DNA-protein complexes was reversed with proteinase K overnight at 65°C. The DNA was purified and then amplified by real-time quantitative PCR with the following primers targeted to *TXNIP* promoter (-615/-502), forward primer: 5^’^-ATCCTCCTTCCACTGGAC-3^’^ and reverse primer: 5^’^-AATGGTTGTTGCGCTCTGG-3^’^; *NOTCH1*-promoter (-737/-584) forward primer: 5^’^-ACACGGCTAGGCCACTCT-3^’^ and reverse primer: 5^’^-ATGTGAATAAGCGGAGGAGCC-3^’^; *SNAI1*-promoter (-171/-42) forward primer: 5^’^- GACAAAGGGGCGTGGCAGATAA-3^’^ and reverse primer: 5^’^-CGCAGAAGAACCACTCGCTA-3^’^.

### Construction of plasmids and transfections

The human *TXNIP* (-2598/+2 bp) promoter was amplified by PCR and inserted into the pGL4.11 luciferase reporter vector (Promega). The putative KLF11 binding site was mutated using the Q5 Site-Directed Mutagenesis Kit (E0554S, New England Biolabs). All PCR products were validated by DNA sequencing. HCAECs were co-transfected with *TXNIP* reporter and Renilla plasmids at 70–80% confluence, using lipofectamine 2000 (ThermoFisher Scientific, 11668019) according to the manufacturer’s protocol. After 24 h, the cells were infected with Ad-*lacZ* and Ad-*KLF11* (10MOI), and 48 h later, the *TXNIP* promoter reporter activity was measured by firefly luciferase (Promega, E1910) and normalized against the control Renilla luciferase activity.

### Endothelial cell preparation and single-cell RNA sequencing

Single-cell suspension of the arterial endothelial cells was prepared as previously described [25, 30]. Briefly, the heart and whole aorta were isolated from *Klf11*^f/f^-*Ldlr*^−/−^ and *Klf11*^ECKO^-*Ldlr*^−/−^ mice fed a DDC (as Control and KO respectively) for 12 weeks, followed by careful removal of the adventitial layer of the aorta under a dissecting microscope. The tissues were subsequently dissected into small pieces and incubated in digestion solution [DMEM (ThermoFisher Scientific, 21063-045), 2 mg/ml collagenase I (Worthington Biochemical Corporation, LS004196), 60 U/ml DNase I (Roche Diagnostics, 1010459001)] at 37 °C for 30–45 min with shaking. Arterial ECs were enriched from cell suspension using CD31 MicroBeads (Miltenyi Biotec, 130097418) according to the manufacturer’s instructions. The enriched cells were then stained with Viable dye eFluor 450 (ThermoFisher Scientific, 50-112-8817) and subjected to FACS to exclude dead cells and sort the viable cells. A sample containing 50% dead cells (heat-killed) was used as a positive control for the viability dye during FACS sorting. The sorted viable cells were subsequently used for scRNA-seq.

The scRNA-seq was performed using Standard 10x Chromium Single Cell 3’ Solution v3.1 (10X Genomics) protocols, and libraries were sequenced on Illumina NovaSeq 6000. The sequencing depths among the two groups were 276,821 mean reads and 1,297 median genes per cell for the Control group; 242,338 mean reads and 1,385 median genes per cell for the KO group. The fraction reads in cells for each sample was > 78%. The raw scRNA-seq data were processed using the 10X Genomics Cell Ranger Software to generate the matrix data. Seurat 4.0 was used for quality control, normalization, clustering, and gene expression profiling.

### Statistical analyses

Statistical analyses were performed using GraphPad Prism 9.0 software (GraphPad Software, San Diego, CA) or RStudio (for scRNA-seq). All quantitative data are presented as Mean ± SEM. All data were first evaluated for normality and variance. For normally distributed data, Student’s *t*-test was used for comparisons between two groups, and one-way ANOVA followed by Tukey’s post hoc analysis or two-way ANOVA followed by Bonferroni post hoc analysis was applied for comparisons among three or more groups. Nonparametric Mann-Whitney test was used for data not normally distributed. *P* value < 0.05 was considered as statistically significant. All results are representative of at least 3 independent experiments.

## Results

### KLF11 is increased in the endothelium of atherosclerotic thoracic aorta

**Fig. 1:**
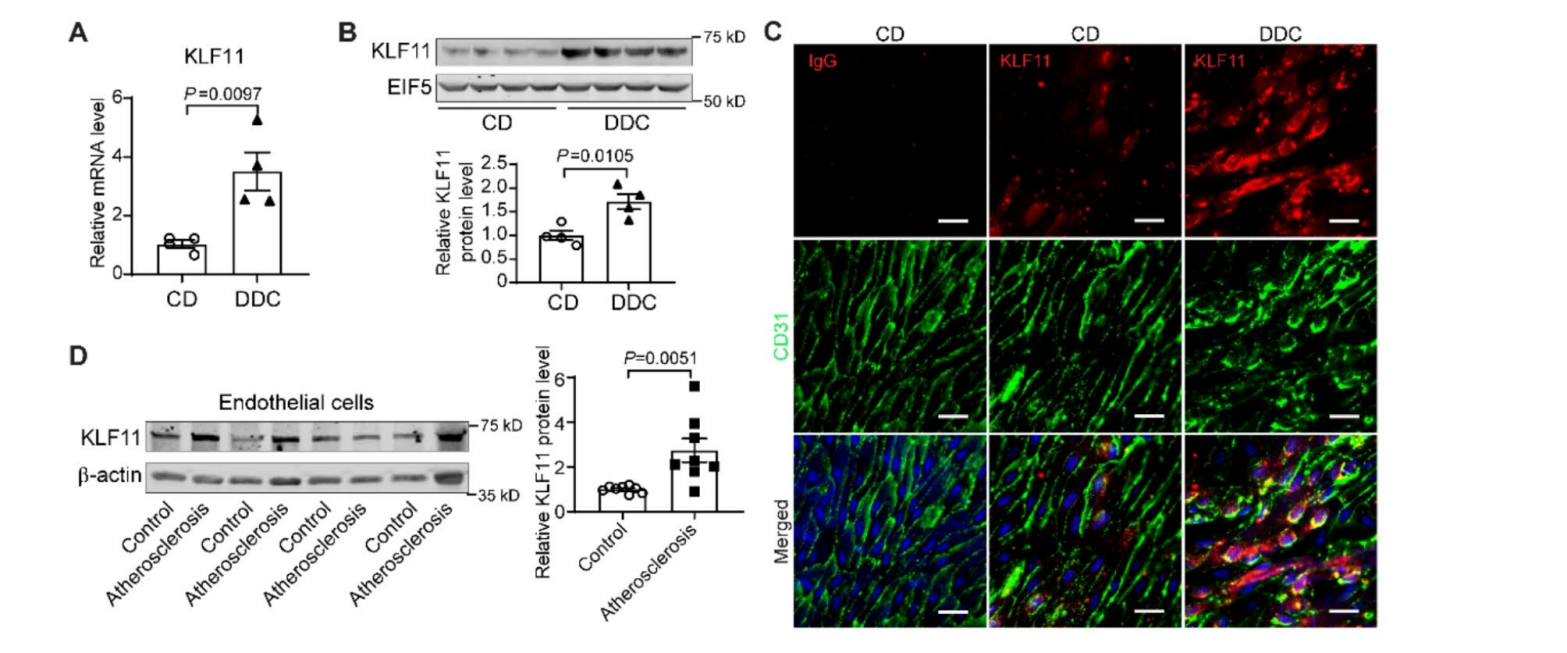
KLF11 is increased in endothelial cells in diabetic atherosclerosis. **A**-**C**, 8-week-old, male, *Ldlr*^−/−^ mice were fed a chow diet (CD) or diabetogenic diet with cholesterol added (DDC, carbohydrate 37.7%, fat 35.7% as lard, protein 17.7%, and 0.15% cholesterol, by weight) for 16 weeks (*n* = 11 mice/group). Aorta and arteries (including the ascending aorta, aortic arch, brachiocephalic left common carotid, and left subclavian arteries) were collected for analysis of KLF11 expression by qPCR (**A**, *n* = 4 mice/group) and Western blot (**B**, *n* = 4 mice/group). **C**, *En face* immunofluorescence staining of KLF11 (Red) and CD31 (green, endothelial cell marker) in the endothelium of ascending aorta. *n* = 3 mice/group. Nuclei stained by DAPI are blue. Scale bar = 20 μm. **D**, Representative western blot analysis of KLF11 expression in the endothelial cells collected from human ascending aortas (*n* = 8 donors/group). Control, samples without plaques from donors with glucose < 125 mg/dL; Atherosclerosis, samples with plaques from donors with glucose > 140 mg/dL. Data are presented as mean ± SEM. P values were calculated using 2-tailed Student’s t-test for A, B, and D

EC dysfunction represents a critical early event in diabetes-related atherosclerosis [11, 31]. In previous studies, we identified KLF11 as a key protector of endothelial activation and dysfunction [18–20]. An analysis of the GEO database (GEO profile: 63666241) indicates that KLF11 is upregulated in vascular ECs of diabetic patients. We induced diabetic atherosclerosis in *Ldlr*^−/−^ mice by feeding a diabetogenic diet added with cholesterol (DDC) for 12 weeks. The DDC-fed mice showed increased body weight, impaired glucose tolerance, and insulin sensitivity, as well as increased lipid profiles compared to the mice fed a standard chow diet (CD) (Supplementary Fig. 1). Notably, we observed an elevated expression of KLF11 in the thoracic aortas of DDC-fed *Ldlr*^−/−^ mice (Fig. 1A-B). Moreover, *en face* immunofluorescence of ascending aorta from DDC-fed *Ldlr*^−/−^ mice showed an increase of KLF11 in the endothelium, with a significant decrease in the expression of EC adhesion junctional protein, VE-cadherin (Fig. 1C). Additionally, we noted an increase of KLF11 protein in the EC of thoracic aortic samples with plaques from human patients with higher blood glucose levels (Fig. 1D and Supplementary Table 1).

### Endothelial KLF11 deficiency aggravates atherosclerosis under diabetic conditions

**Fig. 2:**
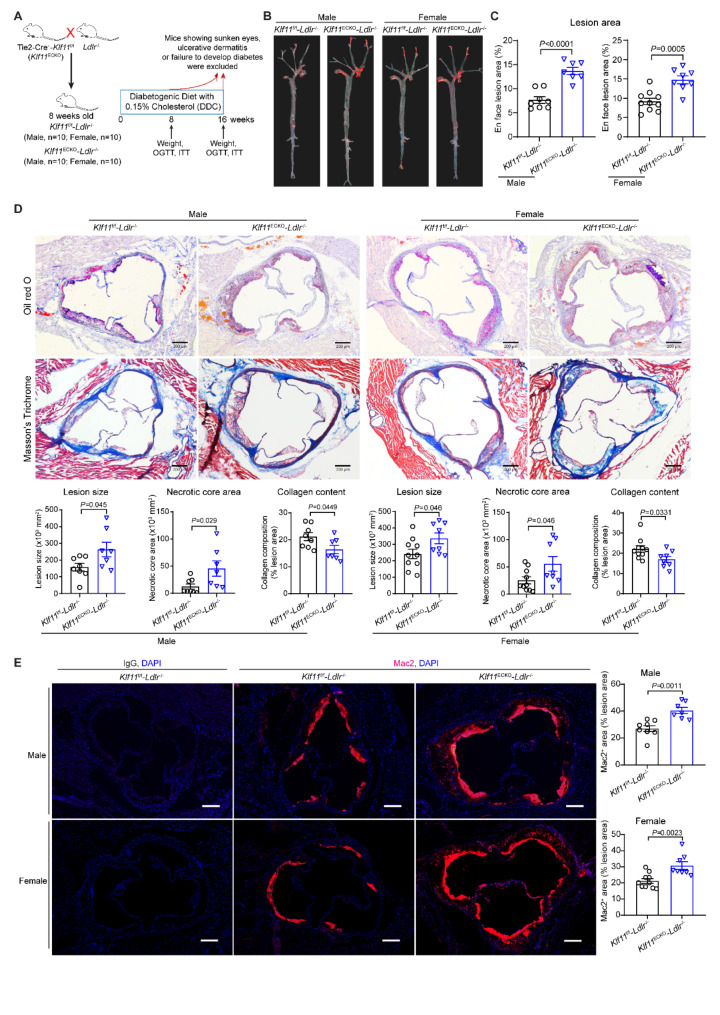
EC-specific KLF11 deficiency aggravates diabetic atherosclerosis. Eight-week-old male and female EC-specific Klf11 knockout (*Tie2Cre*^*+*^-*Klf11*^*f/f*^, *Klf11*^ECKO^) and their littermate floxed-*Klf11* (*Klf11*^*f/f*^) mice bred onto the *Ldlr*^−/−^ background (*Klf11*^ECKO^-*Ldlr*^−/−^ and *Klf11*^f/f^-*Ldlr*^−/−^) were fed DDC for 16 weeks. After the implementation of exclusion criteria, we ended up with *n* = 8 for male *Klf11*^f/f^-*Ldlr*^−/−^ mice, *n* = 7 for male *Klf11*^ECKO^-*Ldlr*^−/−^ mice, *n* = 10 female *Klf11*^f/f^-*Ldlr*^−/−^ mice, *n* = 8 for female *Klf11*^ECKO^-*Ldlr*^−/−^ mice. **A** Schematics of the generation of *Klf11*^ECKO^-*Ldlr*^−/−^ and *Klf11*^f/f^-*Ldlr*^−/−^ mice, DDC feeding for 16 weeks and mice selection. **B**-**C**, Atherosclerotic plaques were stained with Oil Red O and assessed in the aortic trees of those mice. **D**, Atherosclerotic plaques in the aortic sinuses of those mice were stained with Oil Red O and Masson’s Trichrome staining and used for quantification of lesion size, necrotic core area, and collagen content. Scale bar = 200 μm. **E**, Representative immunofluorescence staining and quantification of macrophage (Mac2^+^) infiltration in the plaques of aortic sinuses. Nuclei stained by DAPI are blue. Scale bar = 200 μm. Data are presented as mean ± SEM. P values were calculated using Mann-Whitney test for male data in C, necrotic core area of male mice, and collagen content of female mice in D, or 2-tailed Student’s t-test for all the remaining data in B-E

To investigate the specific role of endothelial KLF11 in diabetic vasculature, both male and female *Klf11*^ECKO^-*Ldlr*^−/−^ and littermate control *Klf11*^f/f^-*Ldlr*^−/−^ mice were subjected to a DDC for 16 weeks (Fig. 2A). At the endpoint, the body weight, glucose tolerance, insulin sensitivity, blood insulin level, lipid profiles and inflammatory cytokine MCP-1 were comparable between *Klf11*^ECKO^-*Ldlr*^−/−^ and *Klf11*^f/f^-*Ldlr*^−/−^ mice (Supplementary Fig. 2). However, both male and female *Klf11*^ECKO^-*Ldlr*^−/−^ mice showed more severe atherosclerosis under diabetic conditions (Fig. 2B-C), assessed by *en face* Oil Red O (ORO) staining of the aortic tree. Moreover, in both male and female *Klf11*^ECKO^-*Ldlr*^−/−^ mice, there was a significant increase in plaque area in the aortic root, accompanied by increases in necrotic cores and macrophage accumulation and a decrease in collagen deposition within the plaques (Fig. 2D-E).

Female *Ldlr*^−/−^ mice have been reported to develop more severe atherosclerosis than their male counterparts [32]. The sex-based disparities in plaque burden and components are also observed in the *Ldlr*^−/−^ mouse model of diabetic atherosclerosis. In our study, we found that female mice were more susceptible to atherogenesis under diabetic conditions compared to their male counterparts, as evidenced by a 19.6% increase in plaque burden in the aortic tree in the control group (*Klf11*^f/f^-*Ldlr*^−/−^, female vs. male, 9.17%±0.816% vs. 7.67%±0.675%) and an 8.1% increase in the knockout group (*Klf11*^ECKO^-*Ldlr*^−/−^, female vs. male, 14.77%±1.008% vs. 13.66%±0.753%) (Fig. 2B-C). Consistently, female *Klf11*^ECKO^-*Ldlr*^−/−^ and littermate control mice showed larger plaque area in the aortic root than their male counterparts (Fig. 2D), while collagen deposition was comparable between male and female mice. Conversely, the content of macrophages in the plaques showed a slight increase in male mice compared to female mice, likely due to the greater plaque area observed in the aortic root in the female mice (Fig. 2E).

### KLF11 overexpression in ECs prevents atherosclerosis under diabetic conditions

**Fig. 3:**
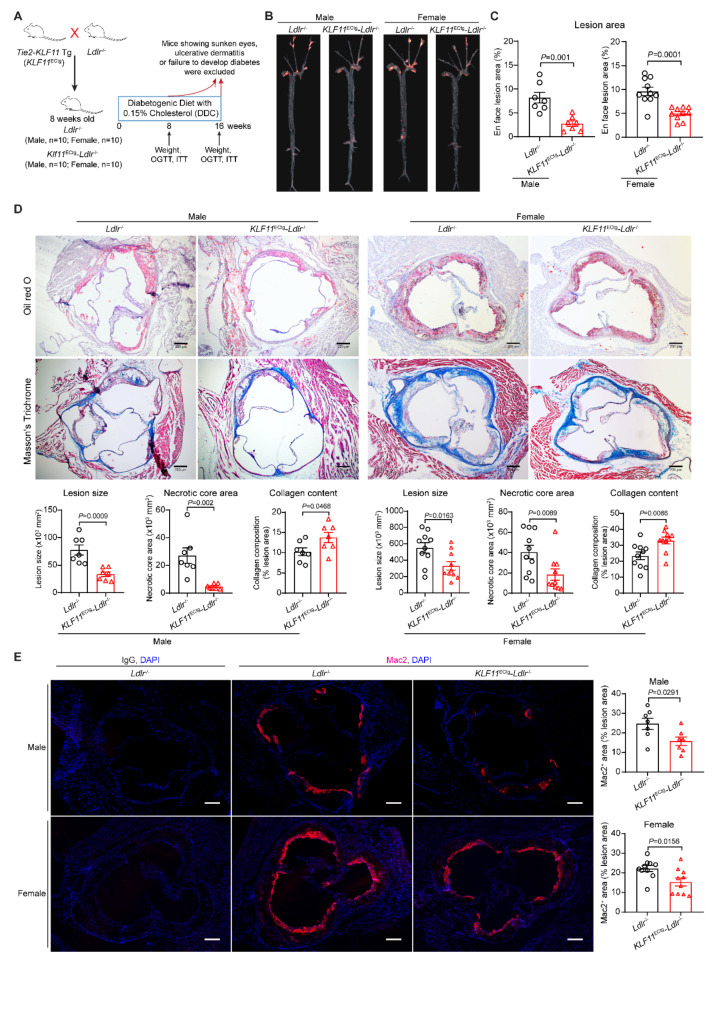
EC-selective overexpression of KLF11 attenuates diabetic atherosclerosis. Eight-week-old male and female EC-selective KLF11 transgenic mice (*Tie2-KLF11* Tg, *KLF11*^ECtg^) and their littermates bred onto a *Ldlr*^−/−^ background (*KLF11*^ECtg^-*Ldlr*^−/−^ and *Ldlr*^−/−^) were fed DDC for 16 weeks. After the implementation of exclusion criteria, we ended up with *n* = 7/group for male mice, *n* = 10/group for female mice. **A** Schematics of the generation of *KLF11*^ECtg^-*Ldlr*^−/−^ and *Ldlr*^−/−^ mice, DDC feeding for 16 weeks and mice selection at endpoint. **B**-**C** Atherosclerotic plaques were stained with Oil Red O and assessed in the aortic trees of those mice. **D** Atherosclerotic plaques in the aortic sinuses of those mice were stained with Oil Red O and Masson’s Trichrome staining and used for quantification of lesion size, necrotic core area, and collagen content. Scale bar = 200 μm. **E** Representative immunofluorescence staining and quantification of macrophage (Mac2^+^) infiltration in the plaques of aortic sinuses. Nuclei stained by DAPI are blue. Scale bar = 200 μm. Data are presented as mean ± SEM. P values was calculated using Mann-Whitney test for necrotic core area of female mice in D or 2-tailed Student’s t-test for all the remaining data in B-E

To further investigate the protective role of endothelial KLF11 in diabetic atherosclerosis, we induced diabetic atherosclerosis in both male and female *KLF11*^ECtg^-*Ldlr*^−/−^ and *Ldlr*^−/−^ mice as described above (Fig. 3A). KLF11 overexpression in ECs did not affect the body weight, glucose tolerance, insulin sensitivity, blood lipid profiles, and MCP-1 in both male and female mice (Supplementary Fig. 3). Nevertheless, atherosclerotic plaque burden in the aortic tree and plaque area in the aortic root were significantly decreased in male and female *KLF11*^ECtg^-*Ldlr*^−/−^ mice (Fig. 3B-D). Moreover, EC-KLF11 overexpression reduced necrotic cores and macrophage accumulation, while increased collagen content in the plaques in both male and female mice (Fig. 3D-E). Furthermore, all female mice developed more severe atherosclerosis under diabetic conditions compared to their male counterparts, showing an increase of plaque burden in the aortic tree both among the control groups (*Ldlr*^−/−^, female vs. male, 9.63%±0.864% vs. 8.18%±1.128%) and transgenic groups (*KLF11*^ECtg^-*Ldlr*^−/−^, female vs. male, 4.99%±0.578% vs. 2.72%±0.578%) (Fig. 3B-C). Notably, EC-KLF11 overexpression showed a more pronounced protective effect in male mice, as evidenced by a 66.7% decrease in plaque burden in the male group (*KLF11*^ECtg^-*Ldlr*^−/−^ vs. *Ldlr*^−/−^, 2.72%±0.578% vs. 8.18%±1.128%) compared to a 48.2% decrease in the female group (*KLF11*^ECtg^-*Ldlr*^−/−^ vs. *Ldlr*^−/−^, 4.99%±0.578% vs. 9.63%±0.864%) (Fig. 3B-C). Concomitantly, male *KLF11*^ECtg^-*Ldlr*^−/−^ mice showed a greater reduction in necrotic cores within the plaques (82.6% decrease in males) compared to females (54.5% decrease in females) (Fig. 3D). Collectively, these results indicate the protective role of endothelial KLF11 against atherogenesis under diabetic conditions and suggest a potential sex-related disparity in endothelial KLF11 functions between males and females.

### KLF11 inhibits EC inflammatory activation in response to high glucose

**Fig. 4:**
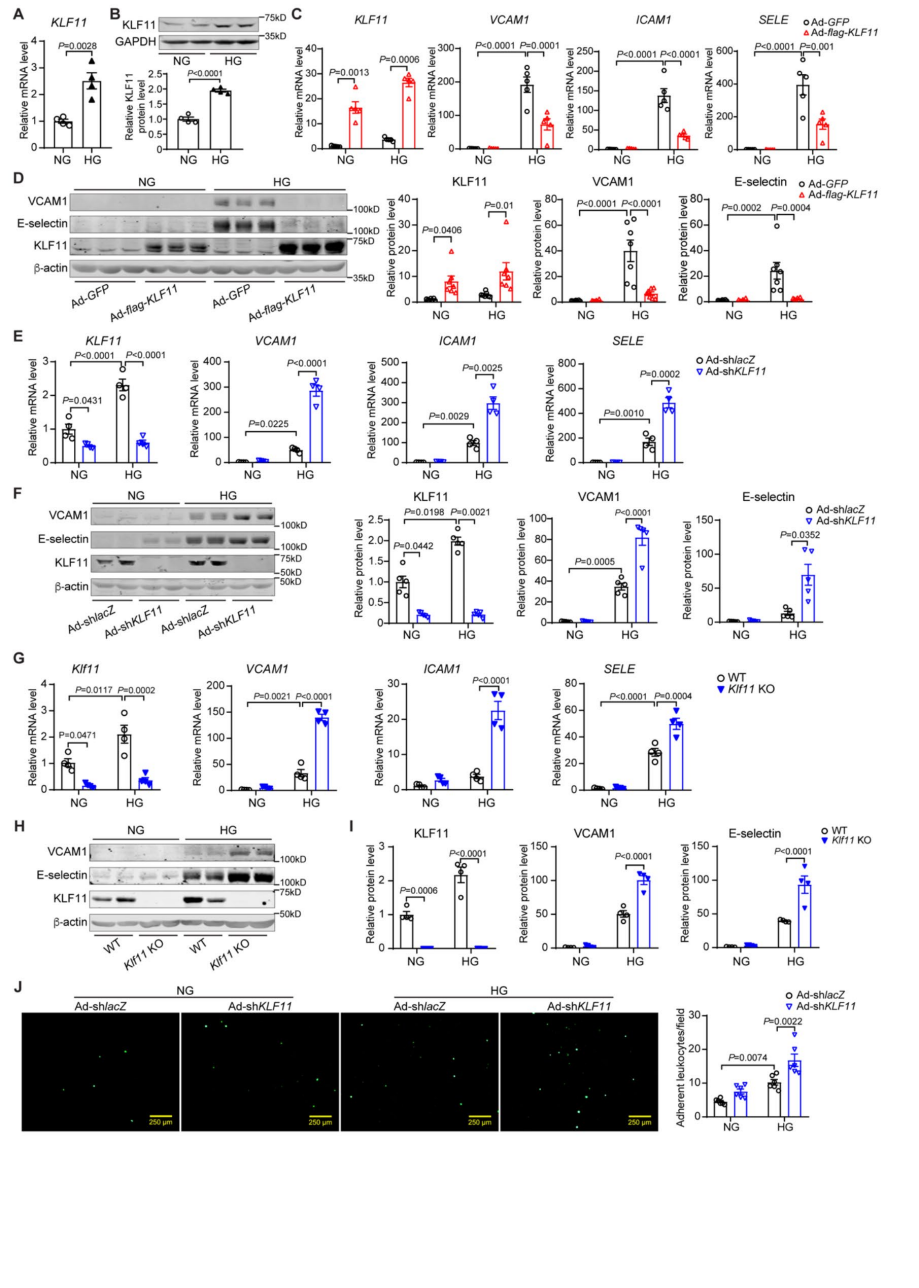
KLF11 is a glucose-inducible transcription factor and inhibits EC inflammatory activation. **A**-**B** Human coronary artery endothelial cells (HCAECs) were treated with high glucose (25 mM D-glucose, HG) for 24 h (A) or 48 h (B). NG, normal level of D-glucose (5mM). qPCR (A) and Western blot (B, representative blots) analysis of KLF11 expression. *n* = 4 samples/group. **C**-**F**, HCAEC were infected with Ad-flag-*KLF11* (10MOI) for KLF11 overexpression or adenovirus carrying-short hairpin RNA for *KLF11* knockdown (Ad-sh-*KLF11*, 10MOI), with Ad-*GFP* and Ad-sh-*lacZ* as control. After 24 h, the cells were treated with HG for 24 h (C, E) or 48 h (D, F). The mRNA of KLF11, VCAM1, ICAM1, and E-selectin was determined by qPCR (C, E) and protein abundance by Western blot (D, F, representative blots). *n* = 5 samples/group for C and F. *n* = 7 samples/group for D. *n* = 4 samples/group for E. **G-I**, Mouse pulmonary endothelial cells (MPECs) isolated from wild type (WT) and KLF11 conventional knockout mice (*Klf11* KO) were treated with HG for 24 h (G) or 48 h (H-I). qPCR (G) and Western blot (H-I, representative blots) were used to analyze the expression of KLF11, VCAM1, ICAM1, and E-selectin. *n* = 4 samples/group. **J**, HCAECs were infected with Ad-sh-*KLF11* (10MOI), Ad-sh-*lacZ* and, 24 h later, treated with HG for 24 h. The activated HCAECs were incubated for 30 min with THP-1 cells labelled with Calcein A/M (1 μm). The binding of THP-1 cells to HCAECs was visualized by fluorescence microscopy and quantified by counting 9 random fields per well using Image J software. Scale bar = 200 μm. *n* = 6 samples/group. Data are presented as mean ± SEM. P values were calculated using 2-tailed Student’s t-test for A-B, or 2-way ANOVA with Bonferroni post hoc multiple-comparison test for C-G and I-J

Endothelial cell activation and dysfunction are initial steps in atherogenesis [10, 33], with their severity further aggravated in the presence of diabetes [12, 34, 35]. In human coronary artery endothelial cells (HCAECs), we found that high glucose (25mM D-glucose, HG) upregulates KLF11 expression at both the mRNA and protein levels (Fig. 4A-B), indicating KLF11 as an inducible transcription factor in response to elevated glucose levels in ECs. Moreover, HG-induced significant elevations of VCAM1, ICAM1, and E-selectin (encoded by *SELE*) in HCAECs, which were suppressed by adenovirus-mediated KLF11 overexpression (Ad-flag-*KLF11*) but aggravated by KLF11 knockdown with adenovirus carrying-short hairpin RNA (Ad-sh-*KLF11*) (Fig. 4C-F, Supplementary Fig. 4A-B). The increased expression of VCAM1, ICAM1, and E-selectin was further confirmed in *Klf11*-deficient mouse pulmonary artery endothelial cells (MPAECs) exposed to HG (Fig. 4G-I). Next, we performed in vitro and in vivo leukocyte-EC adhesion assays. Knockdown of KLF11 and treatment with HG in HCAECs significantly increased THP-1 adhesion to ECs in vitro (Fig. 4J). Moreover, leukocyte rolling and adhesion on the activated ECs in vivo were significantly increased in *Klf11*^ECKO^ mice following intraperitoneally administration with LPS (50 µg/kg) (Supplementary Fig. 4C). These data suggest KLF11 serves as a glucose-inducible transcription factor that counteracts HG-induced EC pro-inflammatory activation by modulating endothelial expression of adhesion molecules.

### KLF11 suppresses EC oxidative stress by transcriptional inhibition of TXNIP

**Fig. 5:**
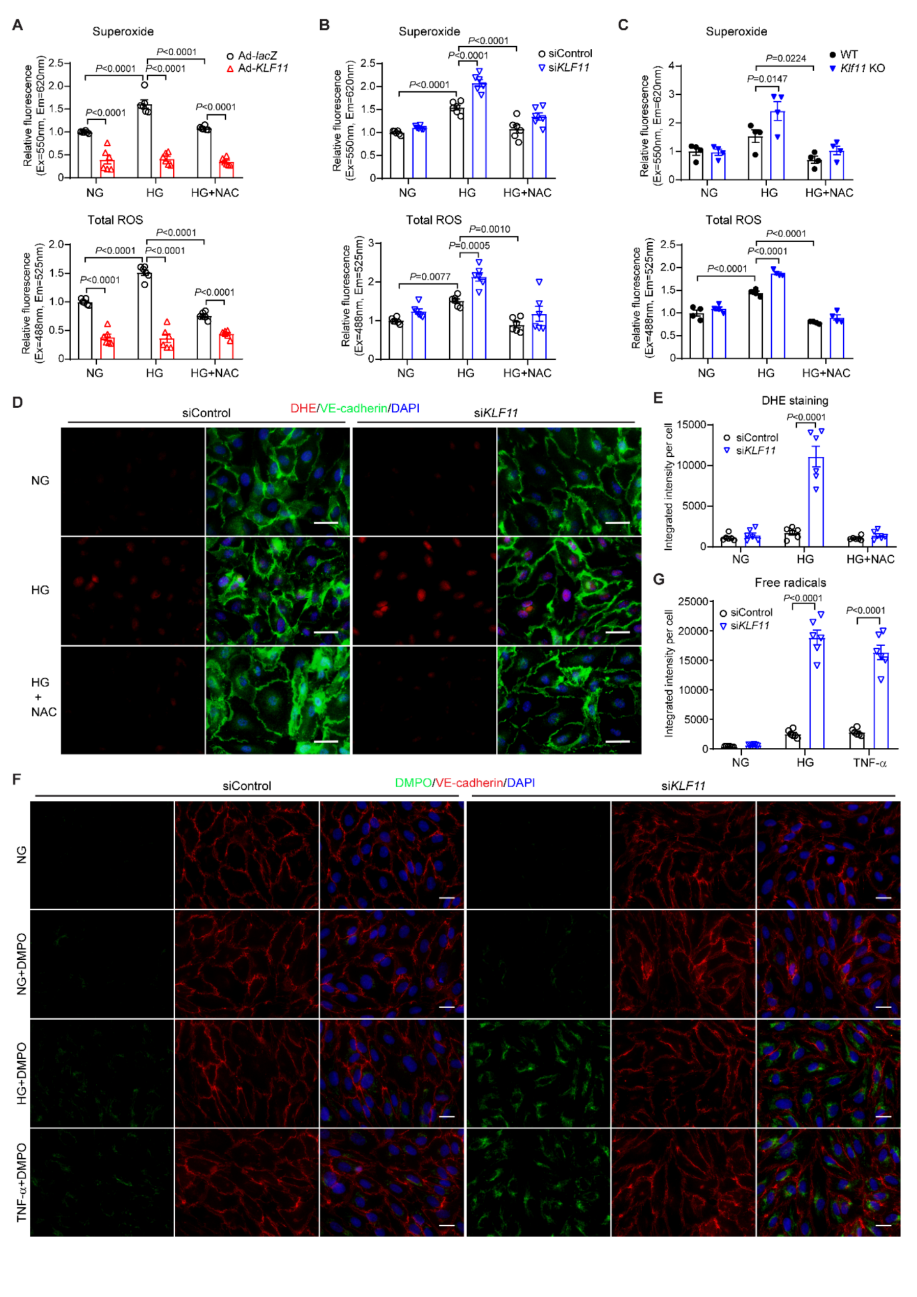
Knockdown of KLF11 enhances EC oxidative stress. **A-C** The production of superoxide and total ROS were examined in HCAECs infected with Ad-*lacZ*, Ad-*KLF1* (A), transfected with control siRNA (siControl), *KLF11* siRNA (si*KLF1*, 20µM) (B), or MPECs isolated from WT and *Klf11* KO (C). After 48 h, the cells were treated with HG for 2 h in the presence of ROS and Superoxide detection reagents. For the negative control, the cells were pre-treated with N-acetyl-L-cysteine (NAC, a ROS inhibitor, 5mM) for 30 min prior to HG induction. *n* = 6 samples/group for A-B. *n* = 4 samples/group for C. **D-G**, HCAECs were transfected with siControl or si*KLF1* (20µM). After 48 h, the cells were treated with HG or TNF-α (2ng/ml) for 2 h, followed by dihydroethidium (DHE) staining (D-E) of superoxide (green) or immune-spin trapping (F-G) to measure the intracellular free radicals (green), in parallel with immunofluorescence staining of VE-cadherin (EC marker). Nuclei stained by DAPI are blue. Scale bar = 20 μm. For negative control in DHE staining, the cells were pre-treated with NAC (5mM) for 30 min prior to HG induction. Cells in NG without DMPO addition was the negative control for the immune-spin trapping staining in F. *n* = 6 samples/group for D-G. Data are presented as mean ± SEM. P values were calculated using 2-way ANOVA with Bonferroni post hoc multiple-comparison test for A-C, E, and G

Excessive reactive oxygen species (ROS) induce oxidative stress, leading to EC dysfunction and atherogenesis [36]. Our data revealed that KLF11 overexpression significantly reduces the production of superoxide and total ROS in HCAECs under both normal and HG conditions (Fig. 5A). Conversely, KLF11 knockdown with siRNA in HCAECs or its deficiency in MPAECs significantly increased superoxide and total ROS levels in ECs exposed to HG (Fig. 5B-C). The increased superoxide in HCAECs upon KLF11 knockdown in the presence of HG was further confirmed by DHE and MitoSOX staining (Fig. 5D-E, Supplementary Fig. 5A-B). Moreover, ROS-caused oxidative DNA damage, assessed by 8-oxo-2’-deoxyguanosine (8-oxo-dG), was also increased in HCAECs following KLF11 knockdown under HG conditions (Supplementary Fig. 5C-D). ROS comprises both free radical and non-free radical oxygen species [37, 38]. Free radicals are highly reactive with other cellular components, such as DNA, proteins, and lipids, which can be quantified by immune-spin trapping with DMPO (5,5-Dimethyl-1-Pyrroline N-oxide) [28, 29, 37, 38]. Accordingly, KLF11 knockdown significantly increased the production of free radicals in HCAECs in response to HG or TNF-α (Fig. 5F-G). These data indicate KLF11 as a protective regulator against EC oxidative stress.

**Fig. 6:**
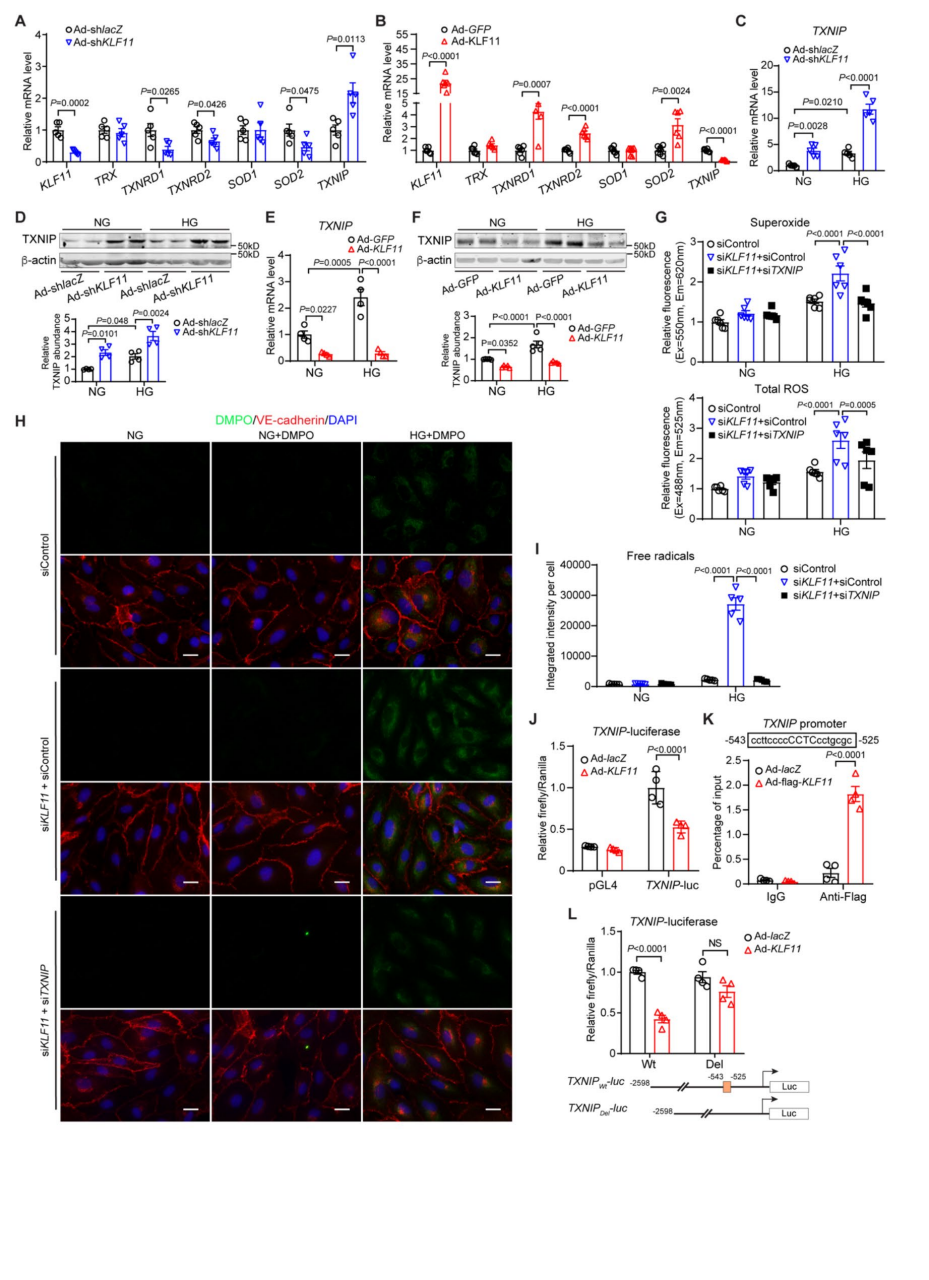
KLF11 attenuates EC oxidative stress through transcriptional inhibition of TXNIP. **A-B**, HCAECs were infected with Ad-sh*lacZ*, Ad-sh*KLF11* for *KLF11* knockdown, or Ad*-GFP*, Ad-*KLF11* for *KLF11* overexpression. After 48 h, the cells were collected for qPCR to determine the mRNA levels of *KLF11*, *TRX* (thioredoxin), *TXNRD1* (thioredoxin reductase 1), *TXNRD2*, *SOD1* (superoxide dismutase 1), *SOD2* and *TXNIP* (thioredoxin interacting protein). *n* = 6 samples/group. **C-F**, HCAECs were infected with Ad-sh*lacZ*, Ad-sh*KLF11*, or Ad*-GFP*, Ad-*KLF11*. After 24 h, the cells were treated with HG for 24 h (C, E) or 48 h (D, F). qPCR (C, E) and Western blot (D, F, representative blots) analysis of TXNIP expression. *n* = 5 samples/group for C. *n* = 4 samples/group for D-E. *n* = 5 samples/group for F. **G**-**I**, HCAECs were transfected with siControl, si*KLF11* or si*KLF11* plus si*TXNIP*. After 48 h, the cells were treated with HG for 2 h. **G**, Superoxide and total ROS were determined in the HCAECs. *n* = 6 samples/group. **H**-**I**, Immune-spin trapping to measure the intracellular free radicals (green). Immunofluorescence staining of VE-cadherin, red. Negative control, NG without DMPO addition. Nuclei stained by DAPI are blue. Scale bar = 20 μm. *n* = 5 samples/group. **J**, HCAECs were transfected with a *TXNIP* promoter (2.5 Kb)-driven luciferase reporter containing a KLF11 binding site and infected with Ad-*lacZ* or Ad-*KLF11*. After 24 h, they were treated with HG for 24 h. The luciferase activity was normalized against that of the co-transfected *Renilla* luciferase reporter. *n* = 4 samples/group. **K**, HCAECs were infected with Ad-*lacZ* or Ad-flag-*KLF11*. After 24 h, they were stimulated with HG for 24 h. Chromatin immunoprecipitation (ChIP) assay was performed using an antibody against Flag or IgG. *n* = 4 samples/group. **L**, HCAECs were transfected with luciferase reporter driven by *TXNIP* promoter, either wild type (Wt) or with KLF consensus-binding site deleted (Del) and then infected with Ad-*lacZ* or Ad-*KLF11.* After 24 h, the cells were stimulated with HG for 24 h. The luciferase activities were examined, and the results are presented relative to cells transfected with Wt and infected with the Ad-*lacZ* group. *n* = 4 samples/group. Data are presented as mean ± SEM. P values were calculated using 2-tailed Student’s t-test for A-B, or 2-way ANOVA with Bonferroni post hoc multiple-comparison test for C-G and I-L

Next, we found that knockdown of KLF11 in HCAECs decreases the expression of the antioxidant enzymes, thioredoxin reductase 1 (TXNRD1), TXNRD2, and superoxide dismutase 2 (SOD2) but upregulates thioredoxin interacting protein (TXNIP) (Fig. 6A). Conversely, KLF11 overexpression showed the opposite effect and significantly downregulated TXNIP expression (Fig. 6B). Furthermore, HG upregulated TXNIP expression in HCAECs (Supplementary Fig. 6A-B), which was further elevated by KLF11 knockdown, but significantly suppressed by KLF11 overexpression (Fig. 6C-F). In the subsequent rescue experiments, we found that the increased production of superoxide, total ROS, and free radicals in HCAECs upon KLF11 knockdown and high glucose exposure was completely abolished by TXNIP knockdown (Fig. 6G-I and Supplementary Fig. 6C). These findings suggest that KLF11 plays a crucial role in mitigating oxidative stress through its regulation of antioxidant enzymes and TXNIP expression.

KLF11 was initially identified as a transcriptional repressor [39]. To investigate whether it directly regulates *TXNIP* expression, we generated a luciferase reporter driven by a 2.5kb *TXNIP* promoter and found that KLF11 overexpression significantly decreases the luciferase activity in HG-treated HCAECs (Fig. 6J). Bioinformatics analysis revealed a potential KLF binding site (–525 to − 543 bp) upstream of the *TXNIP* transcription start site (Fig. 6K). Next, by using the chromatin immunoprecipitation (ChIP) assay, we confirmed that KLF11 indeed binds to the *TXNIP* promoter (Fig. 6K), which is accompanied by an increase in the repressive histone mark H3K27me3 (Supplementary Fig. 6D). Moreover, deletion of the predicted KLF1-binding site in the *TXNIP-*promoter-driven luciferase reporter significantly dampened the KLF11’s regulatory effect on TXNIP (Fig. 6L) in HG-treated HCAECs. Collectively, these data suggest that KLF11 protects against EC oxidative stress by directly binding to its consensus sequence within the *TXNIP* promoter and inducing transcriptional inhibition.

### Endothelial KLF11 deficiency enhances endothelial to mesenchymal transition during diabetic atherosclerosis

**Fig. 7:**
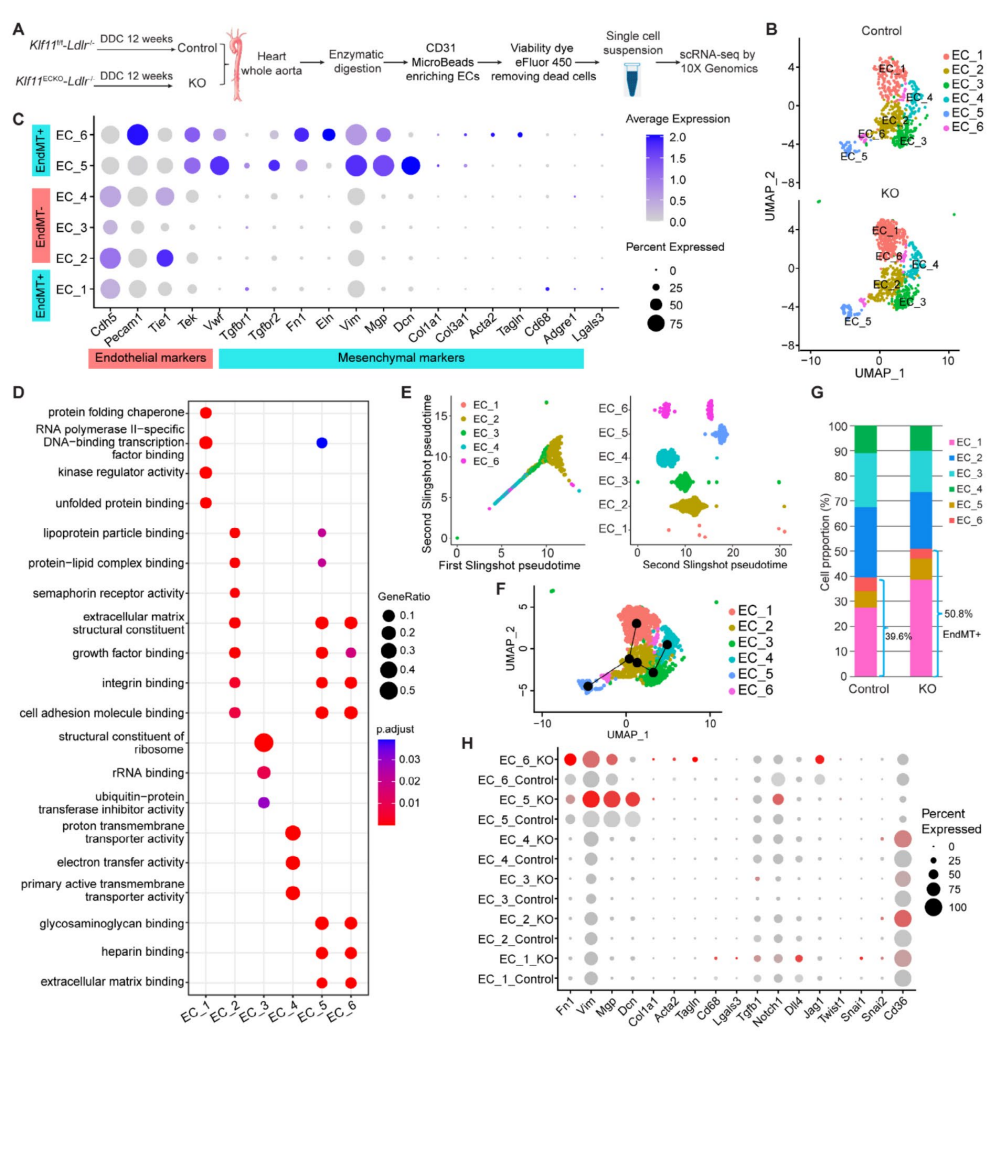
EC-KLF11 deficiency enhances endothelial-mesenchymal transition during diabetic atherosclerosis.** A** Schematic diagram indicating the procedure for scRNA-seq. 8-week-old male *Klf11*^f/f^*-Ldlr*^−/−^ and *Klf11*^ECKO^-*Ldlr*^−/−^ mice were fed a DDC for 12 weeks, termed Control and KO, respectively. The cells isolated from each group were pooled from 3 mice. **B** Uniform Manifold Approximation and Projection (UMAP) plot of EC clusters from Control (*n* = 629) and KO (*n* = 895) groups (n is the cell number qualified for analysis in each group). Colors denote different EC clusters. **C** Dot plot showing the marker genes associated with EC and mesenchymal identity during endothelial to mesenchymal transition (EndMT). EndMT^−^, expressing EC marker and no mesenchymal marker. EndMT^+^, expressing mesenchymal marker with or without EC marker. Size depicts the percentage of cells expressing each gene, and color indicates the expression level. **D** Gene Ontology (GO) enrichment analysis of gene expression in EC clusters. **E**-**F** Trajectory inference with Slingshot. **E** Two-dimensional representation of EC clusters using UMAP with trajectory inferred by Slingshot analysis. **F**, Cells ordered by Slingshot pseudotime. **G** Cell cluster percentages across two experimental groups (Control and KO). **H** Dot plot of EndMT marker genes in EC clusters across the two experimental conditions. Dot size depicts the percentage of cells expressing each gene, and color indicates the expression level

Endothelial to mesenchymal transition (EndMT) drives the initiation and progression of atherosclerosis by accelerating plaque growth and instability [40]. Since KLF11 deficiency in ECs leads to a greater increase in plaque formation in the aortic tree (*Klf11*^ECKO^-*Ldlr*^−/−^ vs. *Klf11*^f/f^-*Ldlr*^−/−^, 78.1% increase in males, 13.66%±0.753% vs. 7.67%±0.675%; 61.1% increase in females, 14.77%±1.008% vs. 9.17%±0.816%) and its overexpression exhibits a stronger protective effect (*KLF11*^ECtg^-*Ldlr*^−/−^ vs. *Ldlr*^−/−^, 66.8% decrease in males, 2.72%±0.578% vs. 8.18%±1.128%; 48.2% decrease in females, 4.99%±0.578% vs. 9.63%±0.864% ) in male mice, we focused on exploring the role of endothelial KLF11 in EndMT during diabetic atherosclerosis and performed scRNA-seq analysis of ECs collected from male *Klf11*^ECKO^-*Ldlr*^−/−^ and *Klf11*^f/f^-*Ldlr*^−/−^ mice fed DDC for 12 weeks, as KO and Control respectively (Fig. 7A). Following quality control and normalization, unsupervised SNN-based clustering was performed to identify cell types (Supplementary Fig. 7A-B and Online Table III). With a focus on ECs, we extracted six clusters of ECs based on the expression of *Cdh5*, *Pecam1*, *Tek*, or *Vwf* (Supplementary Fig. 7C, Fig. 7B, and Online Table IV). Among them, clusters EC_1, EC_5, and EC_6 showed high expression levels of genes associated with mesenchymal phenotype and extracellular matrix genes, such as *Tgfbr1*, *Fn1*, *Vim*, *Mgp*, *Tagln*, and *Cd68* (Fig. 7C). This observation strongly suggests that EndMT occurs during atherogenesis under diabetic conditions. Accordingly, gene ontology (GO) pathway enrichment analysis showed that EC_5 and EC_6 clusters are enriched for the terms of extracellular matrix remodeling, growth factor, and cell adhesion molecular binding (Fig. 7D), further supporting the notion that EC_5 and EC_6 acquire a mesenchymal state. Moreover, we utilized Slingshot to perform trajectory inference analysis and identified 2 trans-differentiation trajectories initiating from EC_2 (Fig. 7E-F), predicting the potential hierarchy between mesenchymal clusters and mature EC clusters. Compared to the Control group, endothelial KLF11 deficiency significantly increases the cell fraction of EC_1 and EC_5 clusters and upregulates the expression of smooth muscle markers and mesenchymal markers in the clusters of EC_1, EC_5, and EC_6 (Fig. 7G-H). Moreover, Notch signaling (Notch1 and Dll4) and transcription factors associated with EndMT, such as Snai1 and Snai2, exhibited increased expression in KLF11 deficient EC clusters (Fig. 7H). Collectively, these data suggest that KLF11 deficiency in ECs increased the EndMT during atherogenesis under diabetic conditions.

### KLF11 inhibits EndMT under high glucose conditions

To explore the mechanism by which KLF11 regulates the EndMT process, we examined the expression of EndMT markers and related transcription factors in HCAECs upon alteration of KLF11 levels. We found that knockdown of KLF11 significantly increases the expression of *ACTA2* (encoding α-actin), *TAGLN* (encoding SM22α), *FN1* (encoding fibronectin 1), *COL3A1* (encoding type III collagen), *VIM* (encoding vimentin), *SNAI1*, NOTCH1, *DLL4* (encoding Delta-like 4) in HG-treated HCAECs (Supplementary Fig. 8A-B). Conversely, KLF11 overexpression effectively prevents the elevation of these genes under HG condition (Supplementary Fig. 8C-D). Our scRNA-seq data also showed the increases of Nocth1 and Snai1, recognized EndMT markers [41], in KLF11-deficient ECs in diabetic atherosclerosis (Fig. 7H). Bioinformatics analysis revealed potential KLF binding sites within the promoters of the *SNAI1* (-122/-140) and *NOCTH1* (-614/-633) (Supplementary Fig. 8E-F). ChIP assays confirmed the increased KLF11 binding to these sites, accompanied by increases in histone methylations upon KLF11 overexpression in HG-treated HCAECs (Supplementary Fig. 8E-F). These data suggest that KLF11 couples to histone methylations to transcriptionally repress SNAI1 and NOCTH1 expression in HG-treated ECs, thereby inhibiting endothelial to mesenchymal transition in response to high glucose conditions.

## Discussion

Accelerated atherosclerosis in patients with diabetes is a major cause of their morbidity and mortality [13, 42]. EC dysfunction is a hallmark of most conditions that are associated with both diabetes and atherosclerosis [11–14]. However, the underlying mechanisms by which hyperglycemia modulates EC function and contributes to atherogenesis in diabetes remain understudied. In this study, we demonstrated that endothelial KFL11 serves as a novel endogenous protective factor against atherogenesis under diabetes by inhibiting EC dysfunction, including inflammatory activation, oxidative stress, and transition to a mesenchymal phenotype in response to high glucose.

The mechanism underlying the protective effects of endothelial KLF11 involve modulation of plaque composition, including necrotic cores and macrophage accumulation. The local death of foam cells combined with impaired efferocytotic capacity of macrophages promotes necrotic core formation [43]. We also found that a small part of ECs with KLF11 deficiency express higher levels of macrophage markers, indicating potential acquisition of macrophage-like features, which may also become foam cells and contribute to necrotic core formation in diabetic atherosclerosis. In addition, collagen deposition within the plaques was significantly reduced in the KLF11 knockout mice with diabetic atherosclerosis, potentially due to the increased expression of MMP9, which was reported to be regulated by KLF11 in ECs our prior study [20]. While diabetes is widely recognized a significant risk for various cardiovascular diseases, including atherosclerosis, the inverse correlation with aortic aneurysm suggests a potentially multifaceted interaction between diabetes and vascular health [44]. By elucidating the specific pathays through which KLF11 exerts its protective effects on aortic aneurysm and diabetic atherosclerosis, we gain valuable insights into potential therapeutic strategies for managing vascular diseases in both diabetic and non-diabetic populations.

Women with type 2 diabetes have a 50% higher risk of CVDs than men [45]. Female *Ldlr*^−/−^ mice also develop more severe atherosclerosis compared to their male counterparts [32]. Previous genetic studies have shown a significant association between KLF11 gene variants and diabetes [5, 7]. However, the KLF11 variants do not demonstrate sex-based disparities in diabetes and cardiovascular phenotypes. Accordingly, we found that severe atherosclerosis and plaque burden are developed in the female *Ldlr*^−/−^ mice under diabetic conditions, which reflects the sex differences observed in humans and could be an appropriate model to explore sex disparities in diabetic atherosclerosis. Although this study points to KLF11 as potentially responsible for some of the sex-based disparities in diabetic cardiovascular risk, the sex-related mechanism underlying these differences and the specific role of endothelial KLF11 in this context remain to be systematically addressed. Moreover, KLF11 has been reported to activate PPARα and downstream lipid oxidation genes, thus reducing hepatic triglycerides and improving fatty liver phenotype in mice [9]. Nevertheless, endothelial KLF11 overexpression or deficiency did not affect the lipid profile in mice with diabetic atherosclerosis. Interestingly, conventional KLF11 knockout protects against high-fat diet-induced obesity and increases metabolic rate in female mice [8]. Of relevance, our scRNA-seq data uncovered potential metabolic effects of KLF11 on endothelial dysfunction and atherosclerosis. The effect of KLF11 on EC metabolic dysfunction during diabetic atherosclerosis and other metabolic disorders creates potential new avenues for further investigation.

Hyperglycemia-induced endothelial activation, an initial step for immune cell recruitment to the vascular wall, is the major contributor to atherogenesis in diabetes [13, 42, 46]. Here, we demonstrated that endothelial KLF11 overexpression attenuated leukocyte infiltration in the plaques in diabetic *Ldlr*^−/−^ mice and prevented high glucose-induced EC inflammation in vitro. In hyperglycemic conditions, the NF-ΚB pathway is mainly implicated in inflammatory response [47]. We previously identified KLF11 as a suppressor of NF-κB signaling via physical interaction with NF-κB/p65 [19, 20], which may largely account for the inhibitory effect of KLF11 on HG-induced EC inflammation. Activator protein-1 (AP-1), and Janus kinase-signal transducer and activator of transcription (JAK-STAT) also play essential roles in the inflammatory response [48]. Considering our findings, we cannot dismiss the possibility that KLF11 might also suppress EC inflammation through these pathways under hyperglycemic conditions. We also found an upregulation of KLF11 in ECs exposed to high glucose, suggesting that KLF11 may act as a negative feedback mechanism to maintain proper physiological inflammatory response. Activated ECs also express tissue factor (TF), the primary initiator of coagulation [49]. We previously demonstrated that KLF11 transcriptionally inhibits TF expression in ECs and vascular smooth muscle cells [17, 50]. Considering that atherosclerotic plaques have elevated TF, the function of KLF11 in inhibiting TF-initiated thrombus formation after plaque rupture warrants further investigation.

Excessive ROS within the vessel bed is a critical mechanism underlying diabetes-associated cardiovascular complications, including atherosclerosis [35, 36, 51]. In diabetic vasculature, uncontrolled accumulation of ROS, especially in ECs, is a direct consequence of hyperglycemia [51]. Oxidative stress is caused by severe imbalance between ROS formation and antioxidative defenses and is regulated by both oxidant and antioxidant enzymes [36, 37, 52]. Thioredoxin interacting protein (TXNIP), an endogenous inhibitor of thioredoxin (TRX), suppresses TRX activity and promotes oxidative stress in multiple cells, such as mesangial cells, VSMCs, and ECs [53–55]. Additionally, TXNIP is also a glucose-sensitive gene, and its deletion has been shown to attenuate hyperglycemia-induced endothelial dysfunction and VSMC inflammation during atherogenesis [53, 56]. In this study, we provide compelling evidence that high glucose induces TXNIP expression in ECs, and its expression is transcriptionally repressed by KLF11. TXNIP has been reported to promote endothelial inflammation by inducing the activation of NLRP3 (NOD-like receptor family pyrin domain containing 3) inflammasome [57]. The possibility that KLF11 inhibition of TXNIP-induced NLRP3 inflammasome activation may also contribute to its anti-inflammatory effect in ECs in response to glucose remains to be explored. Our previous study elucidated the inhibitory effect of KLF11 on NOX2-induced oxidative stress in ECs [20]. Additionally, high glucose induces endothelial NOX2 activation, ultimately leading to EC oxidative stress [58]. Therefore, the activation of NOX2, or other NOXs, may also contribute to the increased ROS accumulation in KLF11 deficient ECs. As an intracellular antioxidant enzyme, glutathione peroxidase 4 (GPX4) can suppress lipid hydroperoxides, decrease EC damage from oxidized lipids, and inhibit atherosclerosis development [59]. Intriguingly, KLF11 is identified as a transcriptional repressor for GPX4, which stimulates ferroptosis in tumorigenesis [60]. Thus, whether and how KLF11 regulates GPX4 transcription and lipid peroxidation in ECs, probably affecting atherosclerosis under diabetic conditions, is a noteworthy possibility to explore.

Increasing endothelial lineage tracing studies indicate that ECs are major sources for plaque-associated mesenchymal cells through EndMT, losing endothelial characteristics and adopting mesenchymal phenotype [40, 61]. Our previous single-cell RNA sequencing (scRNA-seq) study has identified the presence of EndMT in a mouse model of diabetic atherosclerosis [30]. Loss of EC markers such as VE-cadherin and CD31 can lead to increased vascular permeability and immune cell infiltration. Consistently, we found that endothelial KLF11 deficiency increases macrophage accumulation in the plaques under diabetic conditions. Our scRNA-seq data in this study identified three clusters of ECs undergoing EndMT in EC-specific KLF11 knockout mice on the DDC diet,

exhibit increased fraction and elevation of mesenchymal markers, suggesting that they may increase the bad outcomes in diabetic atherosclerosis. High glucose is a well-documented driver of EndMT in diabetic complications by activating a variety of EndMT-inducing pathways and epigenetic modulations [62]. Accordingly, endothelial KLF11 deficiency in the diabetic atherosclerosis model increased the binding activities of lipoprotein particles and growth factors and the expression of Notch1 and Snai1. As ECs undergo EndMT, their expression of classical endothelial markers progressively decreases, making it challenging to identify and accurately characterize these transitioning cells. Additionally, we recognize that the relatively small sample size and cell number may limit our ability to fully explore the consequences of EC-KLF11 KO in the EndMT process in diabetic atherosclerosis and impact the robustness of our conclusions. To address these limitations, further studies should utilize larger sample sizes and combine single-cell transcriptomics with lineage tracing to more accurately profile and identify the differential effects of KLF11 deficiency on the EC clusters undergoing EndMT, thereby strengthening our conclusions. Another limitation of our scRNA-seq study was only performed on male mice. Given that females show more severe atherosclerosis under diabetic conditions, future research should include both male and female mice to better understand the sex-specific effects of KLF11 on EndMT. Additionally, it will be necessary to dissect the different contributions of KLF11 to atherosclerosis in obesogenic vs. diabetogenic conditions by extending these transcriptomic studies to mice on western diet.

KLF11 regulates its target genes by recruiting the CBP/P300/HAT coactivator or the SNI3A/HDAC corepressor [7, 63]. The interaction of KLF11 with the SNI3/HDAC corepressor is indispensable for its transcriptional repressor activity [63]. KLF11 inhibits genes associated with TGF-β pathways and fibrosis during cardiac and renal fibrosis [64]. In some tumors, KLF11 potentiates TGF-β-induced fibrogenesis and epithelial-to-mesenchymal transition due to the disruption of KLF11-SIN3A interaction [39]. These findings raise the possibility that KLF11 inhibits EndMT, at least partially, by recruiting SNI3/HDAC. It has been shown that glucose increase the histone repression mark H3K27me3 at the TXNIP promoter in diabetic mouse kidneys [65]. Here, we showed that KLF11 transcriptionally inhibits the expression of SNAI1 and NOTCH1, accompanied by increased repressive histone mark H3K27me3 and H3K9me2, respectively in high glucose-stimulated ECs. KLF11 is identified as a druggable suppressor for sarcoma cancer stem cells by restraining YAP/TEAD transcription activity via recruiting SIN3A/HDAC [66]. Future studies could specifically address potential interactions between KLF11 and TGF-β, YAP signaling pathways, and how they regulate EC dysfunction in diabetic atherosclerosis.

KLF2 and KLF4 are well documented to inhibit inflammation and oxidative stress in ECs, and KLF7 also exhibits an athero-protective effect by inhibiting glucose metabolic reprogramming in macrophages [67–69]. We previously identified synergistic effects between KLF11 and KLF2, KLF4 on adhesion molecule expression in TNF-α-stimulated ECs. Although KLF11 alteration does not affect endogenous levels of either KLF2, KLF4, or KLF7 [19, 20], these synergistic effects may also occur in high glucose-induced EC inflammation and oxidative stress. Many well-known drugs, such as cysteinyl leukotriene receptor 1 receptor antagonists, glucagon-like peptide 1 analogs, and angiotensin II type I receptor blockers, were found to have anti-atherosclerotic effects, at least partially through KLF2 or KLF4 mediated anti-thrombotic, anti-inflammatory, and antioxidative activities [63, 67, 68]. KLF11 shares many transcriptional targets and protective functions with KLF2 and KLF4 in ECs, responding to many of the same stimuli [63]. Therefore, further research will be required to explore the potential for simultaneous or specific targeting of KLF11 by those known medications and natural bioactive compounds for treating atherosclerosis and associated disorders.

## Conclusions

In summary, using gain- and loss-of-function strategies, we demonstrated endothelial KLF11 as an endogenous protective factor against diabetic atherosclerosis by suppressing inflammatory activation, oxidative stress, and EndMT in ECs exposed to high glucose. We uncovered that the protective effects of KLF11 operate via inhibition of TXNIP-induced EC oxidative stress and Notch1/Snail-mediated EndMT in response to high glucose, all contributing to diabetic atherosclerosis. These findings enhance our understanding of the roles of KLF11 in the vascular system and suggest that targeted manipulation of KLF11 could hold promise for the development of novel therapies to treat diabetes-related cardiovascular complications.

## Electronic supplementary material

Below is the link to the electronic supplementary material.

Supplementary Material 1

Supplementary Material 2

## Data Availability

The single-cell RNA-sequencing datasets presented in this study are available in the Gene Expression Omnibus repository (GSE248065).

## Abbreviations

KLF11: Krüppel like factor 11
EC: Endothelial cell
DDC: Diabetogenic diet with cholesterol
scRNA-seq: Single cell RNA sequencing
EndMT: Endothelial-to-mesenchymal transition
TXNIP: Thioredoxin-interacting protein
VCAM1: Vascular cell adhesion molecule 1
ICAM1: Intercellular adhesion molecule 1
ORO: Oil Red O
ROS: Reactive oxygen species
HCAECs: Human coronary artery endothelial cells
MPAECs: Mouse pulmonary artery endothelial cells
DMPO: 5,5-Dimethyl-1-Pyrroline-N-Oxide
H3K9me2: Di-Methyl-Histone H3 (Lys9)
H3K27me3: Tri-Methyl-Histone H3 (Lys27)
