# Supplementary material for: Endothelial KLF11 is a novel protector against diabetic atherosclerosis
Source: Cardiovasc Diabetol. 2024 Oct 26;23:381. doi: 10.1186/s12933-024-02473-y (PMC11514907; doi:10.1186/s12933-024-02473-y)
Supplement: Supplementary file 2 — Supplementary Material 2 [file 12933_2024_2473_MOESM2_ESM.docx]

**Supplementary Information**

**Endothelial KLF11 is a novel protector against diabetic atherosclerosis**

Guizhen Zhao^1,2^, Yang Zhao^1^, Wenying Liang^1.3^, Haocheng Lu^1,4^, Hongyu Liu^1^, Yongjie Deng^1^, Tianqing Zhu^1^, Yanhong Guo^1^, Lin Chang^1^, Minerva T. Garcia-Barrio^1^, Y. Eugene Chen^1^, Jifeng Zhang^1^

**Affiliations**: ^1^Frankel Cardiovascular Center, Department of Internal Medicine, University of Michigan Medical Center, Ann Arbor, MI 48109, USA; ^2^Department of Pharmacological and Pharmaceutical Sciences, University of Houston College of Pharmacy, Houston, TX 77204; ^3^Division of Rheumatology, Department of Internal Medicine, University of Michigan Medical Center, Ann Arbor, MI 48109, USA; ^4^School of Medicine, Southern University of Science and Technology, Shenzhen 518055, P.R. China

**Supplementary Figures and Figure Legends**

**
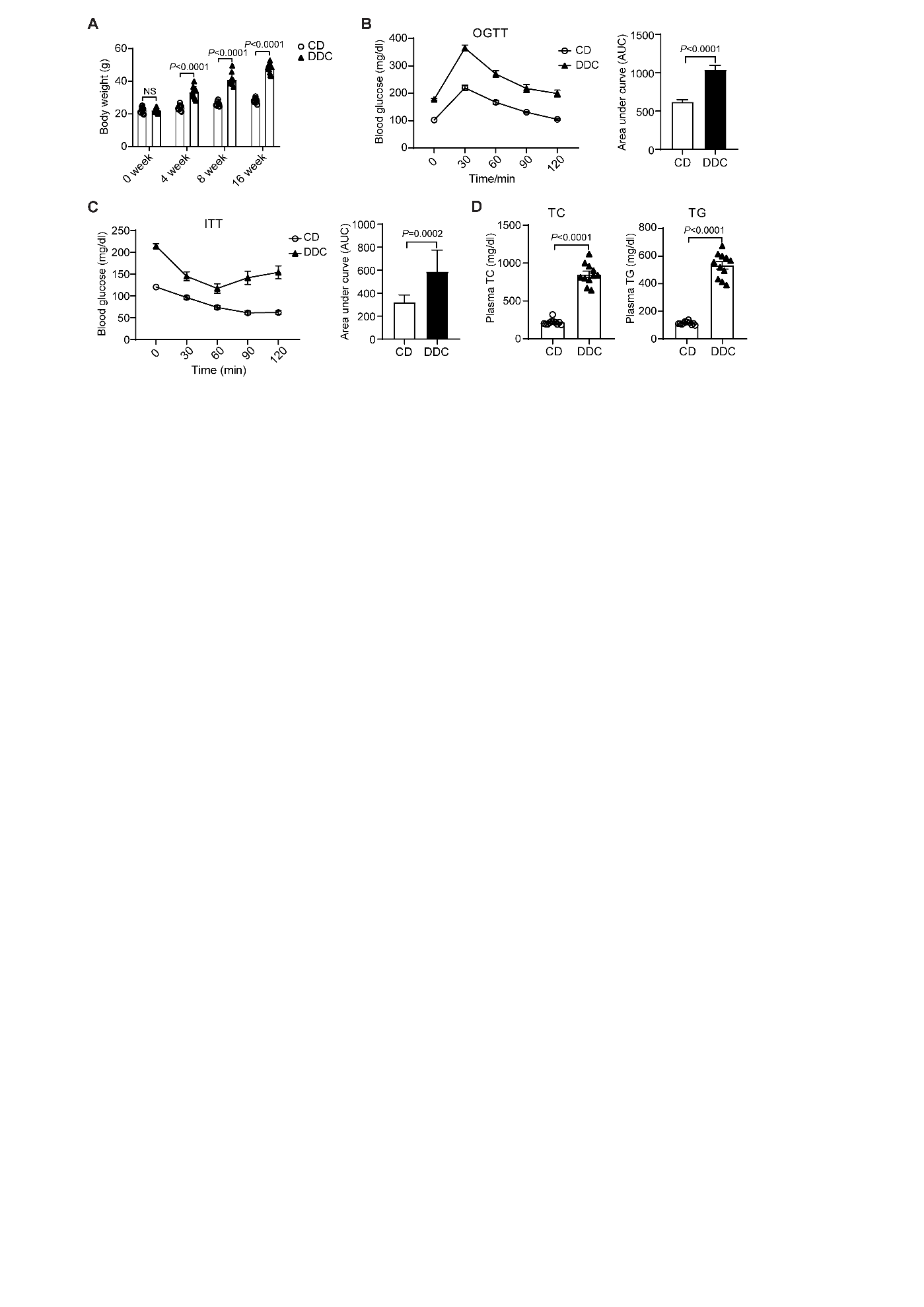
**

**Supplementary Fig. 1. Metabolic changes in *Ldlr*^-/-^ mice fed a diabetogenic diet with cholesterol added**. Eight-week-old male, *Ldlr*^-/-^ mice were fed a chow diet (CD) or diabetogenic diet (carbohydrate 37.7%, fat 35.7% as lard, and protein 17.7%, by weight) with 0.15% cholesterol added (DDC) for 16 weeks. n=11 mice/group. Body weight (**A**), oral glucose tolerance test (OGTT, **B**), insulin tolerance test (ITT, **C**), plasma total cholesterol (TC) and triglyceride (TG) (**D**) after 16 weeks of feeding different diets. n=11 mice/group. Data are presented as mean±SEM. P values were calculated using 2-way ANOVA with Bonferroni post hoc multiple-comparison test for A, or 2-tailed Student’s t-test for B-D.

**
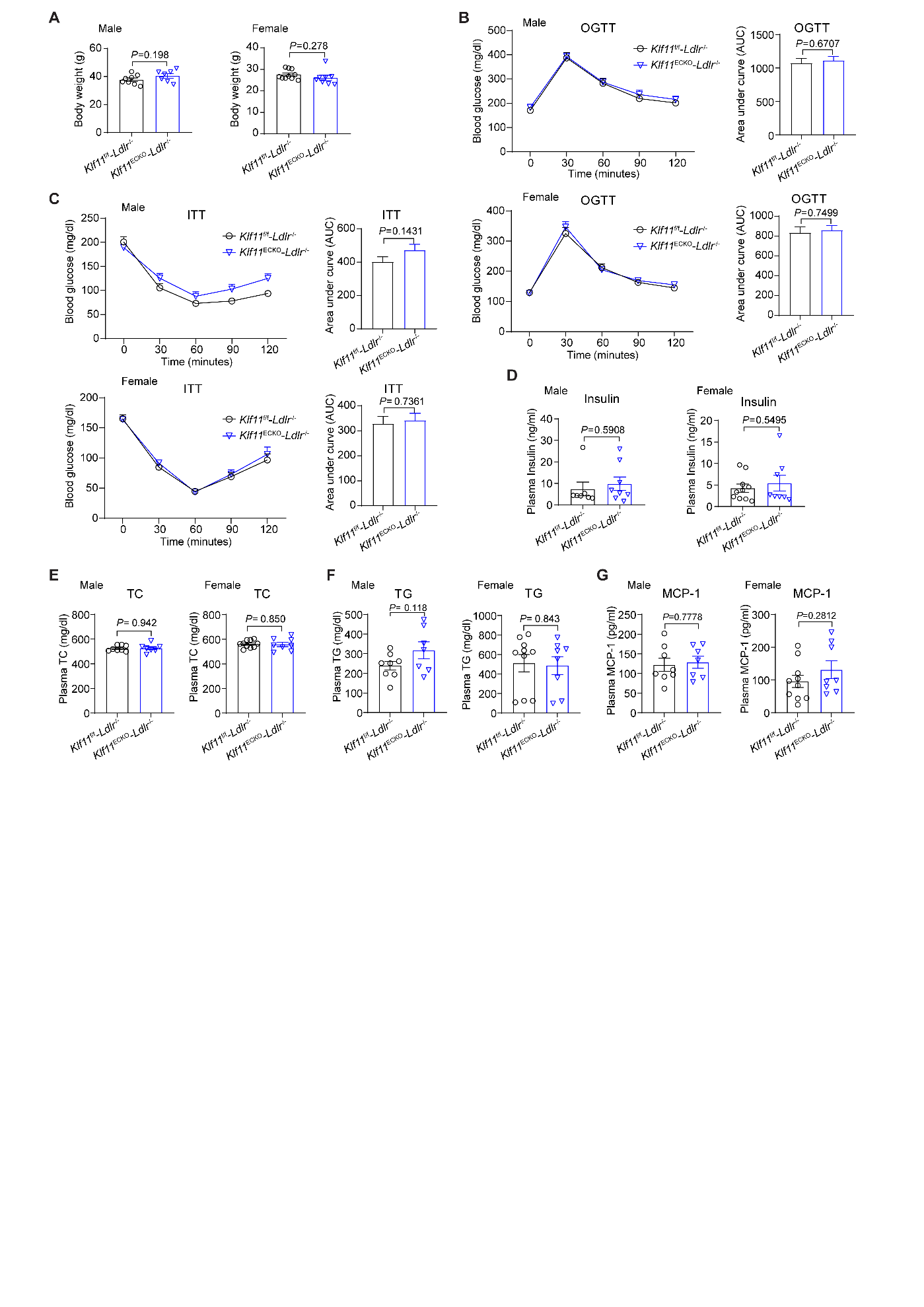
**

**Supplementary Fig. 2. Metabolic profiles of male and female *Klf11*^ECKO^-*Ldlr*^-/-^ and *Klf11*^f/f^-*Ldlr*^-/-^ mice fed DDC.** Eight-week-old male and female *Klf11*^ECKO^-*Ldlr*^-/-^ and *Klf11*^f/f^-*Ldlr*^-/-^ mice were fed DDC for 16 weeks. n=8 for male *Klf11*^f/f^-*Ldlr*^-/-^ mice, n=7 for male *Klf11*^ECKO^-*Ldlr*^-/-^ mice, n=10 female *Klf11*^f/f^-*Ldlr*^-/-^ mice, n=8 for female *Klf11*^ECKO^-*Ldlr*^-/-^ mice. Body weight (**A**), OGTT (**B**), ITT (**C**), plasma insulin (**D**), total cholesterol (**E**), triglycerides (**F**) and monocyte chemoattractant protein-1 (MCP-1, **G**) were determined in the DDC-fed mice. Data are presented as mean±SEM. P values were calculated using 2-tailed Student’s *t*-test for A-G.

**
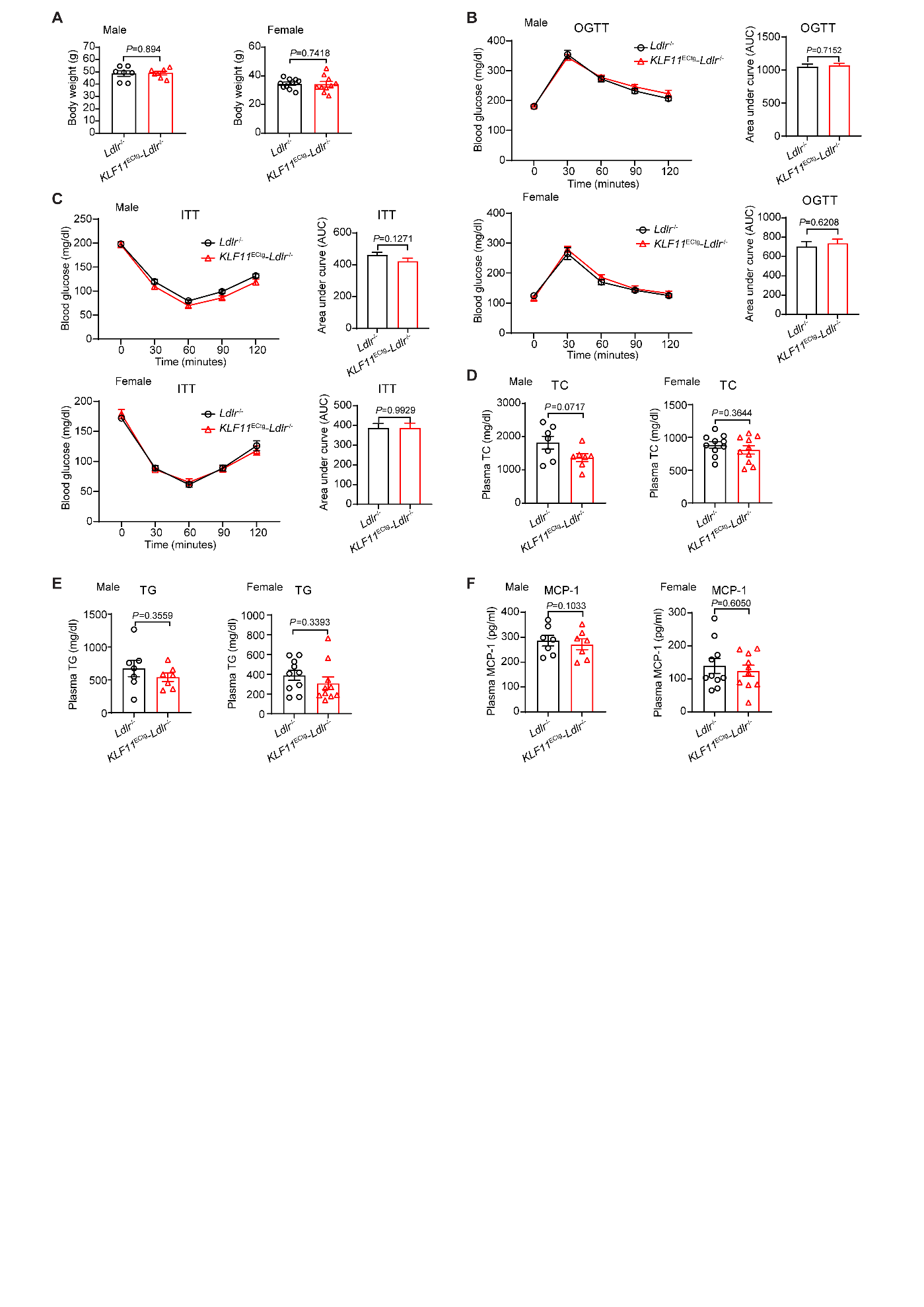
**

**Supplementary Fig. 3. Metabolic profiles of male and female *KLF11*^ECtg^-*Ldlr*^-/-^ and *Ldlr*^-/-^ mice fed DDC.** Eight-week-old male and female *KLF11*^ECtg^-*Ldlr*^-/-^ and *Ldlr*^-/-^ mice were fed DDC for 16 weeks. n=7/group for male mice, n=10/group for female mice. Body weight (**A**), OGTT (**B**), ITT (**C**), plasma total cholesterol (**D**), triglycerides (**E**) and MCP-1 (**F**) were determined in the DDC-fed mice. Data are presented as mean±SEM. P values were calculated using 2-tailded Student’s *t*-test for A-F.

**
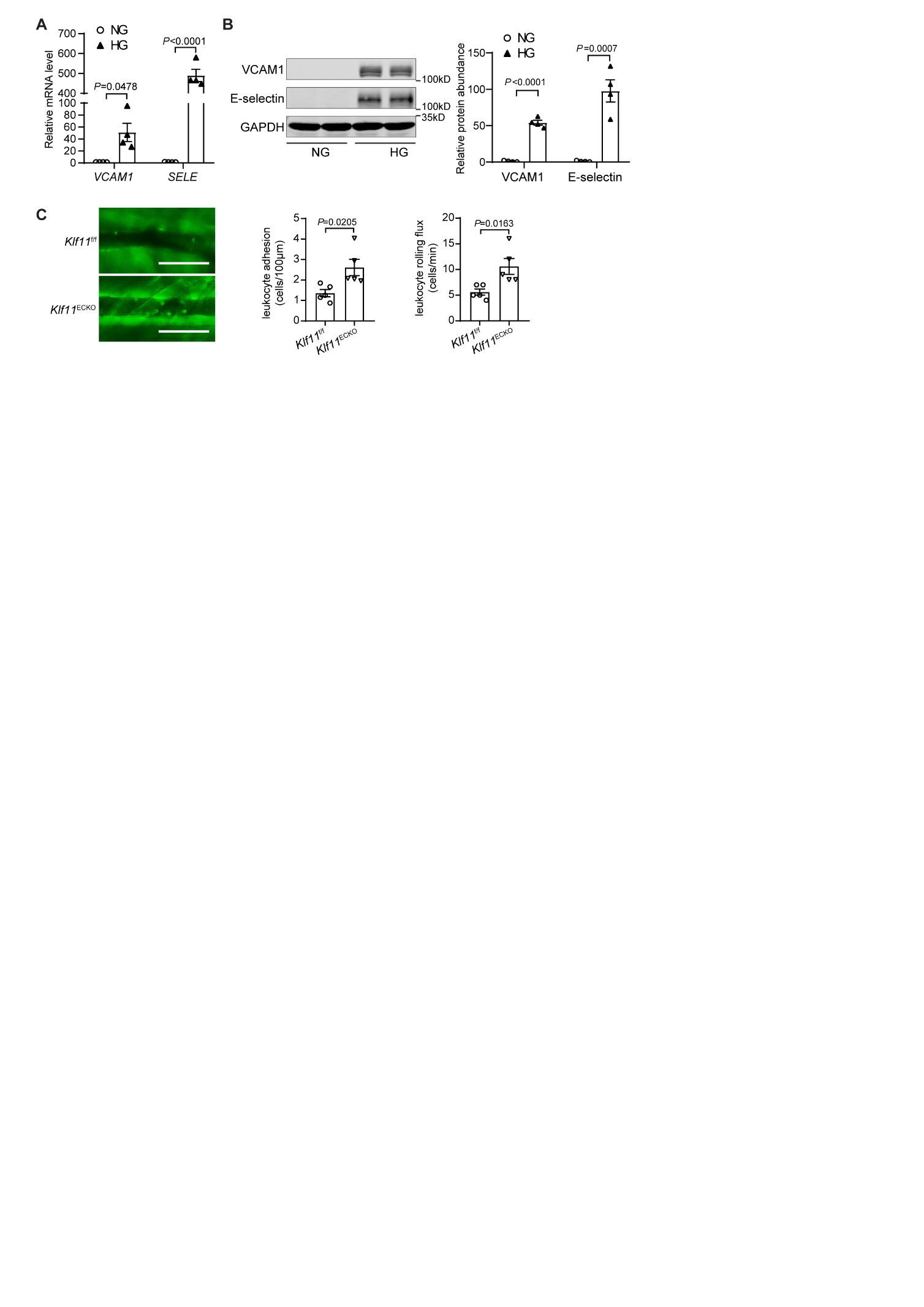
**

**Supplementary Fig. 4. High glucose induces inflammatory response in endothelial cells. A-B**, Human coronary endothelial cells (HCAECs) were treated with high glucose (25 mM D-glucose, HG) for 24h (A) or 48h (B). NG, normal level of D-glucose (5mM). qPCR (A) and Western blot (B, representative blots) analysis of the expression of the pro-inflammatory adhesion molecules VCAM1, and E-selectin. n=4 samples/group. **C**, Male *Klf11*^ECKO^, and *Klf11*^f/f^ mice were intraperitoneally administered with lipopolysaccharide (50μg/kg). After 4h, rhodamine 6G (0.3mg/kg) was infused by tail vein injection to label leukocytes 30min before intravital microscopy analysis. The rolling and adhesion of leukocytes to vascular walls were quantitatively analyzed. Scale bar=200μm. n=6 mice/group. Data are presented as mean±SEM. P values were calculated using 2-tailed Student’s *t*-test for A-C.


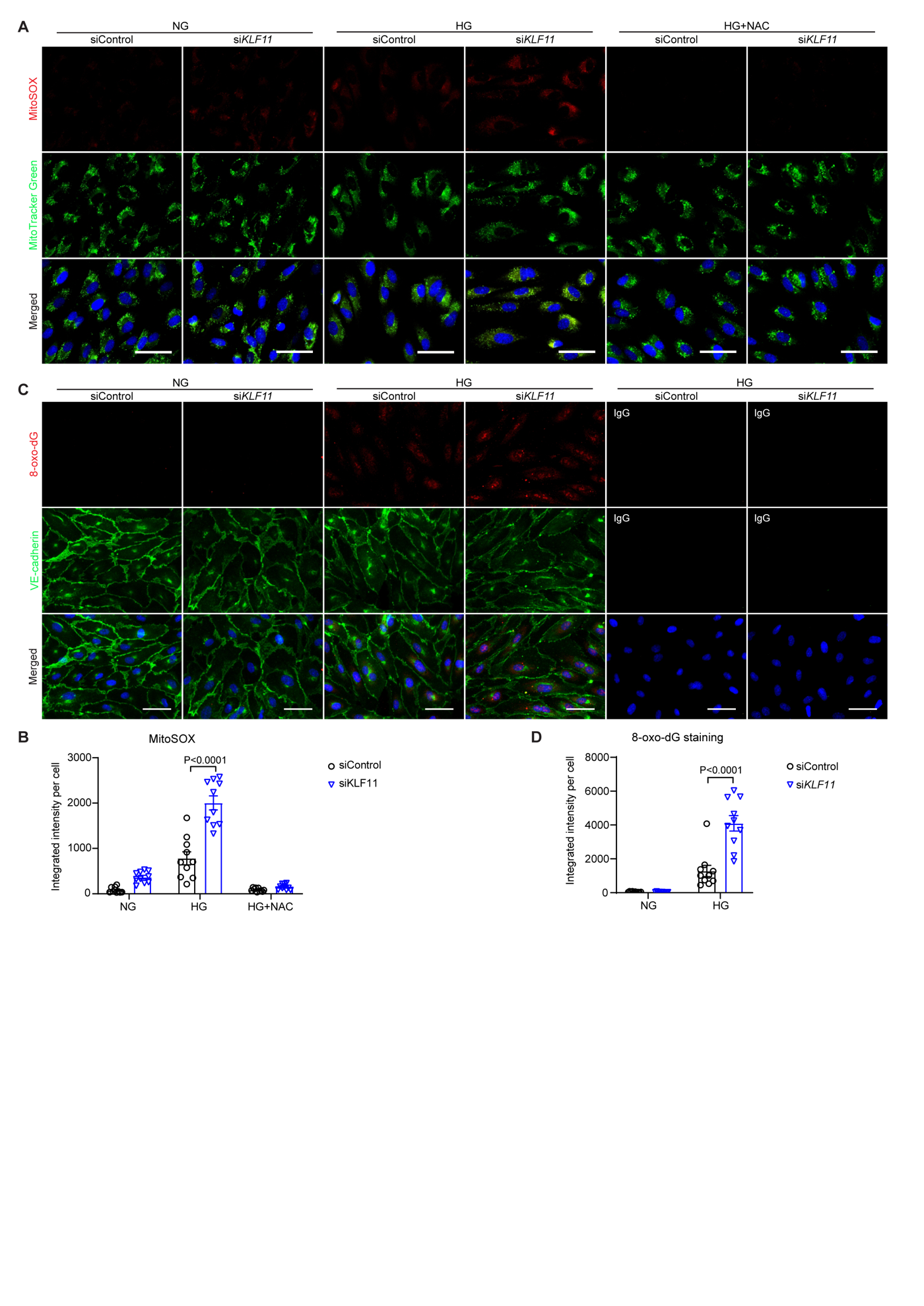


**Supplementary Fig. 5. Knockdown of KLF11 enhances EC oxidative stress.** HCAECs were transfected with siControl or si*KLF11* (20µM). After 24h, the cells were treated with HG for 2h, followed by staining with MitoSOX (red) and MitoTracker (green) (**A**) or labelled with antibody against 8-hydroxy-2’-deoxyguanosine (8-oxo-dG antibody, red) and VE-cadherin (EC marker, green) (**C**). Nuclei stained by DAPI are blue. Scale bar=50µm. For the negative control in MitoSOX staining, the cells were pre-treated with N-acetyl-L-cysteine (NAC, a ROS inhibitor, 5mM) for 30 min prior to HG induction. Quantification of mitochondrial superoxide (**B**) in A and oxidative DNA damage (**D**) in C. n=10 samples/group. Data are presented as mean±SEM. P values were calculated using 2-way ANOVA followed by Bonferroni post hoc multiple-comparison test for B, D.

**
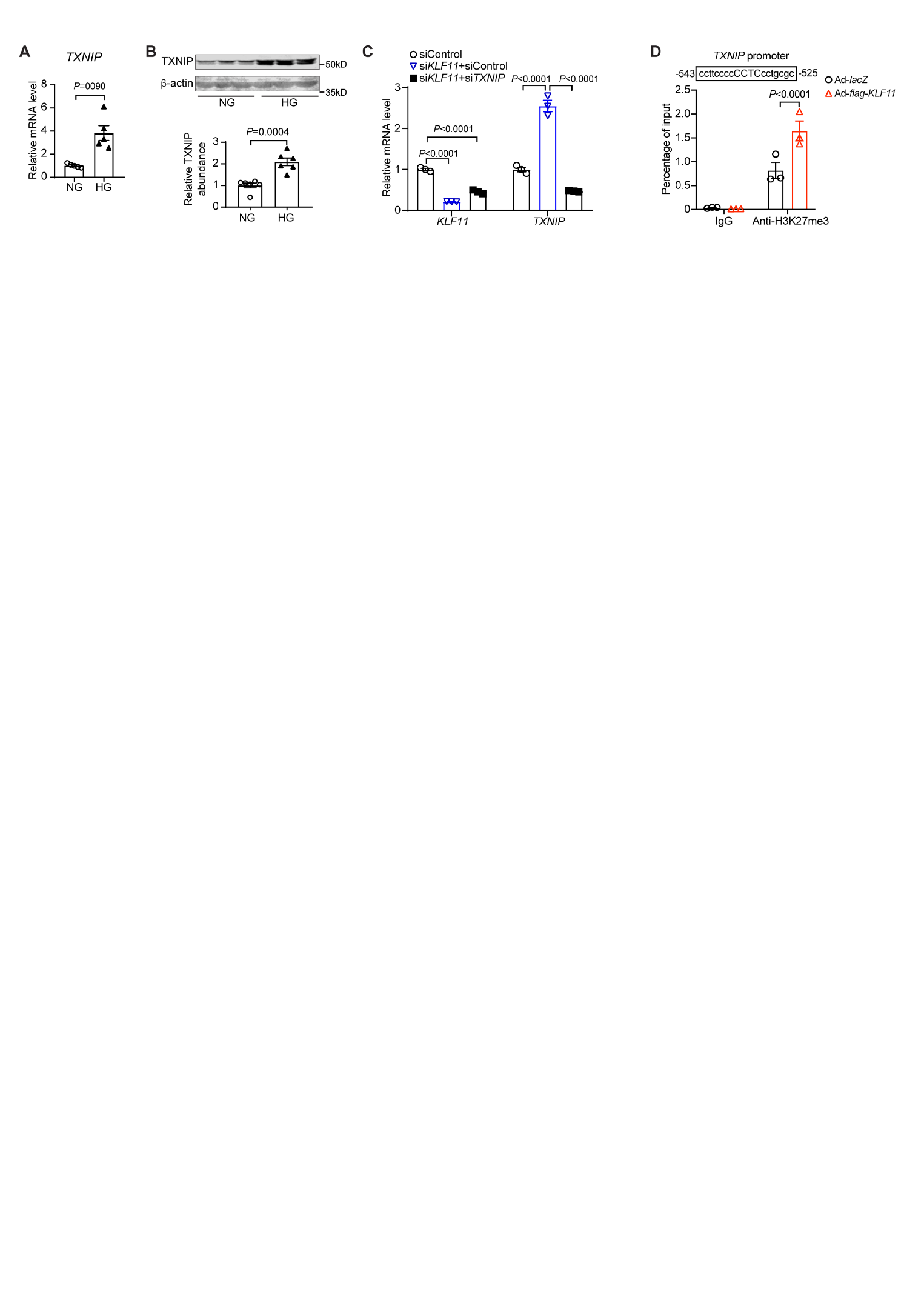
**

**Supplementary Fig. 6. KLF11 inhibits TXNIP1 expression in HG-treated HCAECs. A**-**B**, HCAECs were treated with or without HG for 24h. qPCR (B, n=5 samples/group) and western blot (C, representative blots, n=6 samples/group) analysis of TXNIP expression. **C**, qPCR analysis of KLF11 and TXNIP expression in HCAECs, which were transfected with siControl, si*KLF11,* or si*KLF11* plus si*TXNIP*. n=3 samples/group. **D**, HCAECs were infected with Ad-*lacZ* or Ad-flag-*KLF11*. After 24h, they were stimulated with HG for 24h. Chromatin immunoprecipitation (ChIP) assay was performed using an antibody against H3K27me3, or IgG. n=3/group. Data are presented as mean±SEM. P values were calculated using 2-tailed Student’s *t*-test for A-B, 1-way ANOVA with Tukey’s post hoc multiple-comparison test for C, or 2-way ANOVA followed by Bonferroni post hoc multiple-comparison test for D.

**
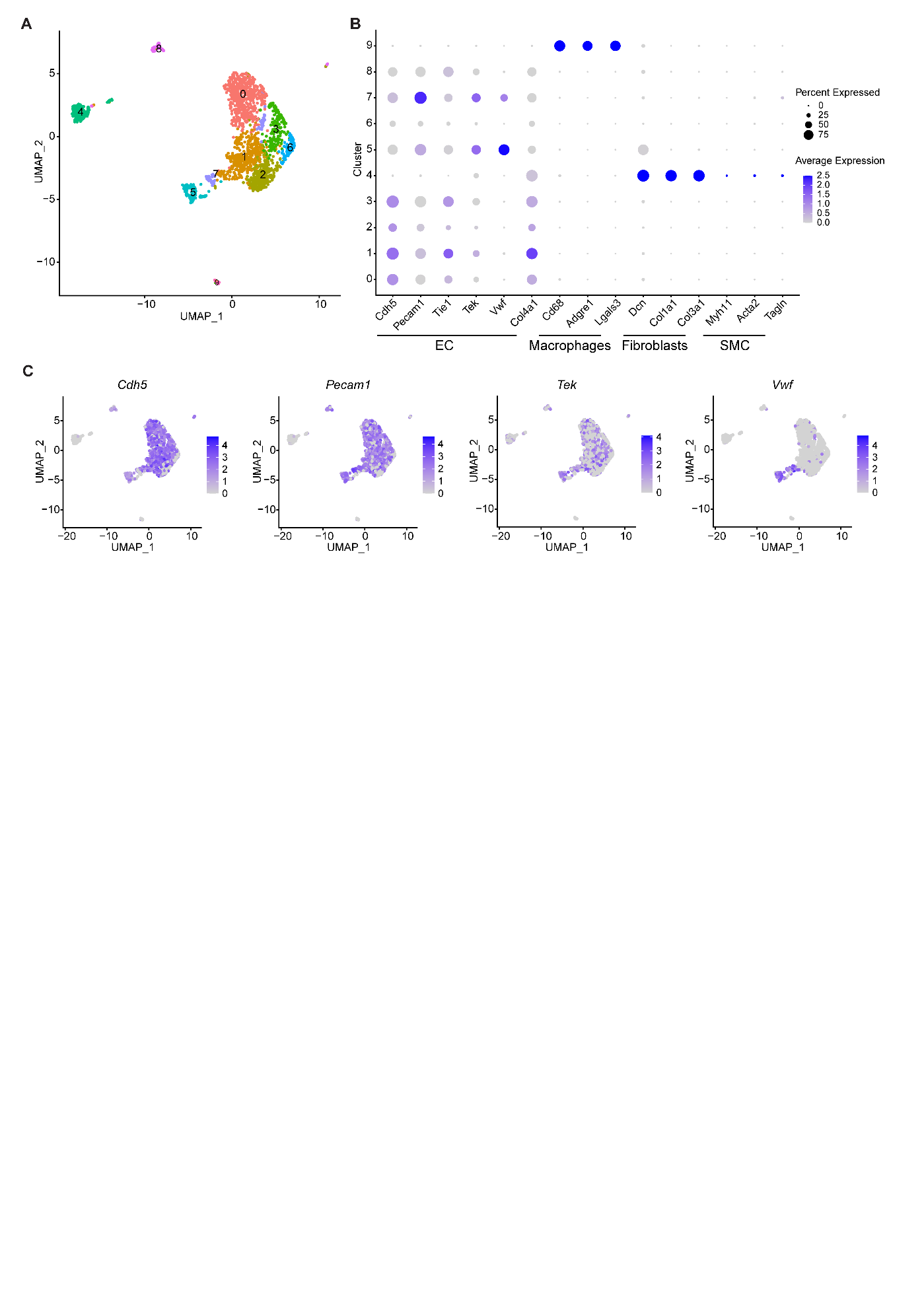
**

**Supplementary Fig. 7. Identification of cell clusters in mouse arteries by single-cell RNA sequencing (scRNA-seq).** 8-week-old male *Klf11*^f/f^*-Ldlr*^-/-^ and *Klf11*^ECKO^-*Ldlr*^-/-^ mice were fed a DDC for 12 weeks, as Control and KO respectively. The single cells isolated from each group were pooled from 3 mice and subjected to scRNA-seq. **A,** Uniform Manifold Approximation and Projection (UMAP) plot of aggregate cells from Control and KO group. Colors denote different cell clusters. After quality control, 770 and 1071 cells from Control and KO were captured for clustering analysis. **B**, Dot plot of selected marker genes for each cluster and lineage. Dot size indicates the percentage of cells expressing each gene, and dot color represents the average expression level. EC, endothelial cells. SMC, smooth muscle cells. **C**, Expression of EC marker genes (*Cdh5*, *Pecam1*, *Tek,* and *Vwf*) was visualized by Feature plots.

**
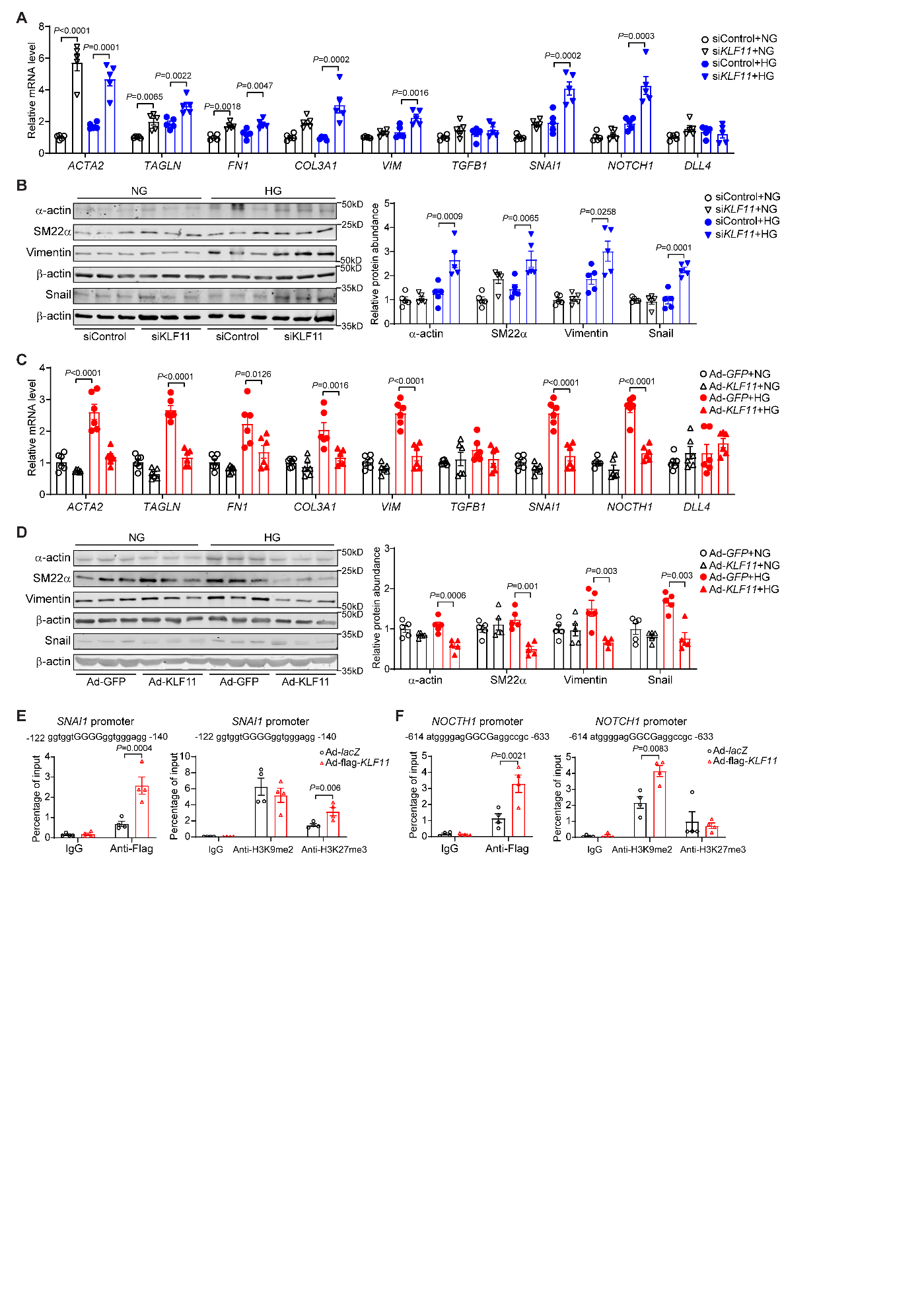
**

**Supplementary Fig. 8. KLF11 regulates EndMT through the TGF-β signaling pathway. A-D**, HCAECs were transfected with siControl, si*KLF11* for *KLF11* knockdown, or Ad*-GFP*, Ad-*KLF11* for *KLF11* overexpression. After 24h, the cells were treated with HG for 48h and collected for qPCR (A, C) of *ACTA2*, *TAGLN*, *FN1*, *COL3A1*, *VIM,* *TGFB1*, *SNAI1*, *NOTCH1*, *DLL4*, and Western blot (B, D, representative blots) of α-actin, SM22α, Vimentin, Snail. n=5/group for A, B and D. n=6/group for C. **E**-**F**, HCAECs were infected with Ad-*lacZ* or Ad-flag-*KLF11*. After 24h, they were stimulated with HG for 24h. Chromatin immunoprecipitation (ChIP) assay was performed using an antibody against Flag, H3K9me2, H3K27me3, or IgG. n=4/group. Data are presented as mean±SEM. P values were calculated using 2-way ANOVA followed by Bonferroni post hoc multiple-comparison test for A-F.
